# Duration‐Dependent Cardiac Effects of Anabolic‐Androgenic Steroid Use in Strength‐Trained Athletes: A Systematic Review and Meta‐Analysis

**DOI:** 10.1111/echo.70611

**Published:** 2026-08-31

**Authors:** Matheus de Medeiros Fernandes, Pedro Gomes Batista, Camila Viviane Ribera Chagas, Railla Raquel Albino dos Santos Silva, Fernanda Medeiros Santos, Miguel Chaves Lenzi, Felipe de Medeiros Fernandes, Ranna Santos Pessoa, Patrícia Jovelina de Freitas, Flávio Henrique Amaral Pires Véras, Stephan Barisic Junior, Douglas Nunes Cavalcante, Juliana Giorgi, Caroline Fischer‐Bacca

**Affiliations:** ^1^ Medicine Department State University of Rio Grande Do Norte (UERN) Mossoró Brazil; ^2^ Medicine Department Federal University of Paraíba (UFPB) João Pessoa Brazil; ^3^ Medicine Department Federal University of Ceará (UFC) Fortaleza Brazil; ^4^ Medicine Department Federal University of Rio Grande Do Norte (UFRN) Natal Brazil; ^5^ Hospital Israelita Albert Einstein Goiânia Brazil; ^6^ Sírio Libanês Hospital São Paulo Brazil; ^7^ Albert Einstein Hospital São Paulo Brazil; ^8^ University Center for the Development of Alto Vale Do Itajaí (UNIDAVI) Rio do Sul Santa Catarina Brazil

**Keywords:** anabolic steroids, athletes, cardiac remodeling, echocardiography, global longitudinal strain

## Abstract

**Purpose:**

Nonmedical anabolic‐androgenic steroid (AAS) use is increasing, and its duration‐dependent cardiac effects remain unclear. We evaluated cardiac structure and function in AAS‐using strength‐trained athletes, overall and by exposure duration.

**Methods:**

We searched PubMed, Embase, Cochrane Central, and SCOPUS through November 2025 for observational studies comparing AAS‐using and nonusing strength‐trained athletes of either sex. Random‐effects models pooled mean differences (MD) overall and across four exposure strata (<6 months, 6 months–3 years, 3–6 years, >6 years); meta‐regression assessed duration as a continuous moderator.

**Results:**

Forty studies (1906 athletes; 95% male) were included. Overall, AAS users showed reduced left ventricular ejection fraction (LVEF; 29 studies; MD −2.84%; 95% CI −4.24 to −1.44; *p* < 0.01), impaired global longitudinal strain (GLS; MD +3.27%; *p* < 0.01), and increased left ventricular mass index (LVMI; MD +18.21 g/m^2^; *p* < 0.01). Septal thickening was detectable before six months, preceding functional impairment. LVEF, LVMI, and posterior wall thickness (PWT) differed significantly across strata (all *p* ≤ 0.03), with the largest LVEF reduction beyond six years (MD −7.39%; 95% CI −10.71 to −4.07). Meta‐regression suggested that exposure duration was associated with variation in selected structural and functional outcomes, corroborating LVEF, LVMI, and PWT, while GLS, E/e′, and E/A ratio showed no significant association. Right ventricular strain was impaired in an exploratory, non‐stratified analysis.

**Conclusion:**

AAS use is associated with cardiac structural and functional abnormalities, with exposure duration associated with selected, but not all, outcomes. These cross‐sectional comparisons do not establish within‐person progression; longitudinal surveillance with deformation imaging is warranted.

## Graphical abstract

Anabolic‐androgenic steroid use is associated with cardiac structural remodeling and functional impairment in strength‐trained athletes. Longer exposure was significantly associated with greater reductions in LVEF and increases in LVMI and posterior wall thickness, while other echocardiographic abnormalities showed heterogeneous temporal patterns. *First draft generated with Claude AI*.

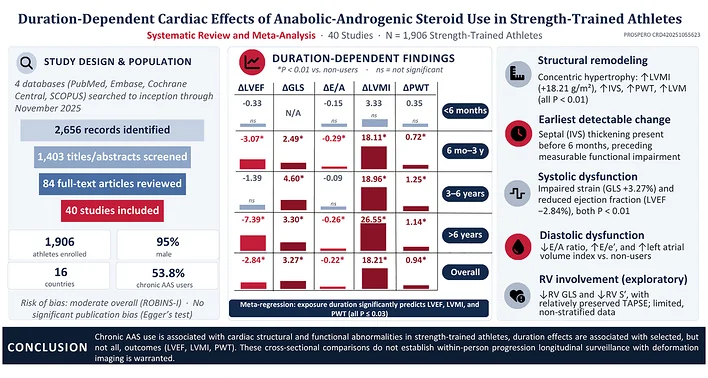

**Abbreviations**
AAS: anabolic‐androgenic steroid
AI: artificial intelligence
CaMKII‐MEF2: calcium/calmodulin‐dependent protein kinase II—myocyte enhancer factor 2
CI: confidence interval
E/A: early‐to‐late ventricular filling ratio
E/E′: E‐wave to E′ ratio
E′: early diastolic mitral annular velocity
GLS: global longitudinal strain
*I* ^2^
: heterogeneity index
IVS: interventricular septal thickness
LA: left atrium
LAVI: left atrium volume index
LV: left ventricular
LVEDV: left ventricular end‐diastolic volume
LVEF: left ventricular ejection fraction
LVESV: left ventricular end‐systolic volume
LVM: left ventricular mass
LVMI: left ventricular mass index
MD: mean difference
mTOR: mammalian target of rapamycin
PeV: peak early diastolic filling velocity
PRISMA: preferred reporting items for systematic reviews and meta‐analyses
PROSPERO: international prospective register of systematic reviews
PWT: posterior wall thickness
REML: restricted maximum likelihood
ROBINS‐I: risk of bias in non‐randomized studies of interventions
RV: right ventricular / right ventricle
RVGLS: right ventricular global longitudinal strain
RVS′: right ventricular systolic velocity
RWT: relative wall thickness
SD: standard deviation
TAPSE: tricuspid annular plane systolic excursion
TTE: transthoracic echocardiogram

## 1 Introduction

Nonmedical use of anabolic‐androgenic steroids (AAS) has been reported since the 1960s to enhance athletic performance [1]. A recent meta‐analysis estimates that the global prevalence of AAS use among athletes may reach 13.4% [2]. Long‐term AAS use is associated with adverse physical outcomes, with mortality rates up to 20‐fold higher than in non‐users, often due to cardiovascular causes [3, 4].

Cardiac structural and functional alterations are consistently described in AAS users, including increased left ventricular (LV) mass and impairment of systolic and diastolic function [5]. While these changes may overlap with physiological athletic remodeling, AAS use appears to shift this adaptation toward a pathological phenotype, potentially increasing the risk of arrhythmias [4, 6].

In a recent meta‐analysis, AAS use among athletes was associated with impairment of LV systolic function and structural cardiac remodeling [7]. However, it did not characterize how these alterations vary according to cumulative exposure time, and meta‐regression did not show significant associations between duration and structural parameters [7]. This suggests that stratified approaches may be necessary to better understand the differences in cardiac patterns across various exposure periods.

Evidence from prospective cohort studies suggests that some early cardiac changes may be reversible. In the HAARLEM study, supraphysiological AAS exposure was associated with increased LV mass and impaired systolic and diastolic function [8]. However, after a mean recovery period of eight months following discontinuation, echocardiographic parameters returned to baseline values. Taken together, these data raise the possibility that AAS‐induced cardiac remodeling follows a dynamic trajectory, with early potentially reversible changes that may progress to persistent dysfunction with prolonged exposure.

Despite these reports, the association between prolonged exposure and cardiac alterations remains incompletely understood. To date, no meta‐analysis has systematically evaluated AAS‐related remodeling using stratified exposure categories. Therefore, this study aimed to characterize the associations between AAS use and cardiac remodeling according to exposure duration, evaluating transthoracic echocardiographic (TTE) parameters across four clinically relevant time‐based strata.

## 2 Materials and Methods

The study followed Preferred Reporting Items for Systematic Reviews and Meta‐Analyses (PRISMA) guidelines and Cochrane recommendations [9, 10]. This systematic review is registered on the International Prospective Register of Systematic Reviews (PROSPERO CRD420251055623) [11].

### 2.1 Eligibility Criteria

Studies were eligible if they met all the following criteria: (1) adult strength‐trained athletes with chronic AAS use, with no a priori restriction by sex; (2) observational design (cross‐sectional, case‐control, or cohort); (3) TTE assessment of LV, right ventricular (RV), or left atrial (LA) structure or function; (4) inclusion of a non‐using strength‐trained comparator group; and (5) reporting of AAS exposure duration or follow‐up time. Exclusion criteria comprised: (1) absence of a control group; (2) case reports, reviews, or conference abstracts without extractable full data; (3) populations with cardiovascular disease unrelated to AAS use; and (4) insufficient data for effect‐size estimation

### 2.2 Search Strategy and Study Selection

We systematically searched PubMed, Embase, Cochrane Central, and SCOPUS from inception to November 1, 2025, without filters or date limits (strategy in Supplementary Material 1). Reference lists of included studies and previous systematic reviews were also manually screened for additional records. Two investigators (M.M.F. and M.C.L.) independently performed the literature search and study selection, with any disagreements resolved by consensus with a third party (J.G.). After duplicate removal, studies underwent a two‐stage screening process: an initial abstract assessment for relevance followed by a thorough full‐text review to confirm eligibility. Final findings and study suitability were compared and adjusted through investigator consensus.

### 2.3 Data Extraction and Outcomes Assessed

Data were independently extracted in duplicate by M.M.F and M.C.L., with discrepancies resolved by consensus and, when unresolved, arbitration by a third investigator (J.G.). Extracted variables included study design, country, sample size, AAS compound and administration pattern, cumulative exposure duration, and all echocardiographic outcomes of interest.

Functional parameters included left ventricular ejection fraction (LVEF), global longitudinal strain (GLS), E/e′, E/A ratio, peak early diastolic filling velocity (PeV), early diastolic mitral annular velocity (E′), left ventricular end‐diastolic volume (LVEDV), and left ventricular end‐systolic volume (LVESV). Structural parameters included left ventricular mass (LVM), left ventricular mass index (LVMI), relative wall thickness (RWT), interventricular septal thickness (IVS), and posterior wall thickness (PWT). RV parameters comprised tricuspid annular plane systolic excursion (TAPSE), RV S′, and RV GLS; LA remodeling was assessed via LA volume index (LAVI).

### 2.4 Exposure‐Duration Classification

AAS users were stratified into four exposure categories based on cumulative lifetime use: <6 months, 6 months – 3 years, 3–6 years, and >6 years. Allocation was determined using a standardized, point‐estimate rule applied to the exposure data reported in each primary study: studies reporting exposure as mean ± SD or median (IQR) were allocated using the mean or median as the central point estimate; studies reporting a range were categorized using the midpoint of the range; and studies reporting only an open‐ended threshold (e.g., “>2 years” or “>5 years”) were assigned using the reported lower bound directly as the most conservative point estimate available, with full study‐level assignments detailed in Supplementary Material 2.

Given that wide standard deviations and open‐ended duration bounds are normative across the AAS echocardiography literature, strict exclusion of studies with wide or overlapping exposure windows was not implemented, as doing so would systematically deplete the evidence base and introduce selection bias. Instead, to account for continuous variability and directly test for a duration–response gradient without relying solely on discrete binning, exposure duration was additionally operationalized as a continuous moderator in random‐effects meta‐regression models for outcomes with sufficient study‐level data.

### 2.5 Statistical Analysis

Pooled estimates are reported first for the overall, non‐stratified analysis, followed by exploratory analyses stratified by exposure duration under time schemes described above. Continuous outcomes were pooled as mean differences (MDs) with 95% confidence intervals (CIs) using an inverse‐variance, random‐effects model with the restricted maximum‐likelihood (REML) estimator for τ^2^. Where studies reported medians, ranges, or interquartile ranges, these were converted to means and SDs using the method of Wan et al. [12]. Statistical significance was defined as a two‐sided *p* value <0.05. Between‐study heterogeneity was quantified using the Cochran Q test and the *I* ^2^ statistic. All analyses were performed in R (version 4.5.0).

Because the majority of primary studies reported AAS exposure duration as a wide mean ± SD or as an open‐ended threshold rather than as a narrow, single‐stratum value, a sensitivity analysis based on strict exclusion of studies whose exposure distribution could span more than one category was not performed; such an exclusion would have removed a large, non‐random share of the evidence base, since studies with broad or open‐ended exposure reporting are systematically different from those with narrow, well‐defined exposure windows, risking a selection bias at least as serious as the misclassification bias it was intended to address.

Instead, as our primary safeguard against exposure misclassification, random‐effects meta‐regression was performed using each study's point‐estimate exposure duration as a continuous moderator, for outcomes with sufficient study‐level data (LVEF, GLS, and LVMI), and formally quantified between‐study heterogeneity with the Cochran Q test for each pooled and subgroup estimate.

### 2.6 Quality Assessment

The risk of bias in each study was assessed independently by two authors (R.R.A.S. and C.V.R.C.) using Cochrane's tools for assessing risk of bias in non‐randomized studies (ROBINS‐I) [13]. Disagreements were resolved by arbitration by a third party (J.G. and M.M.F.). A funnel plot to assess publication bias was generated in R and analyzed using visual inspection and Egger's test, following the Cochrane Handbook [9].

## 3 Results

### 3.1 Study Selection

**FIGURE 1:**
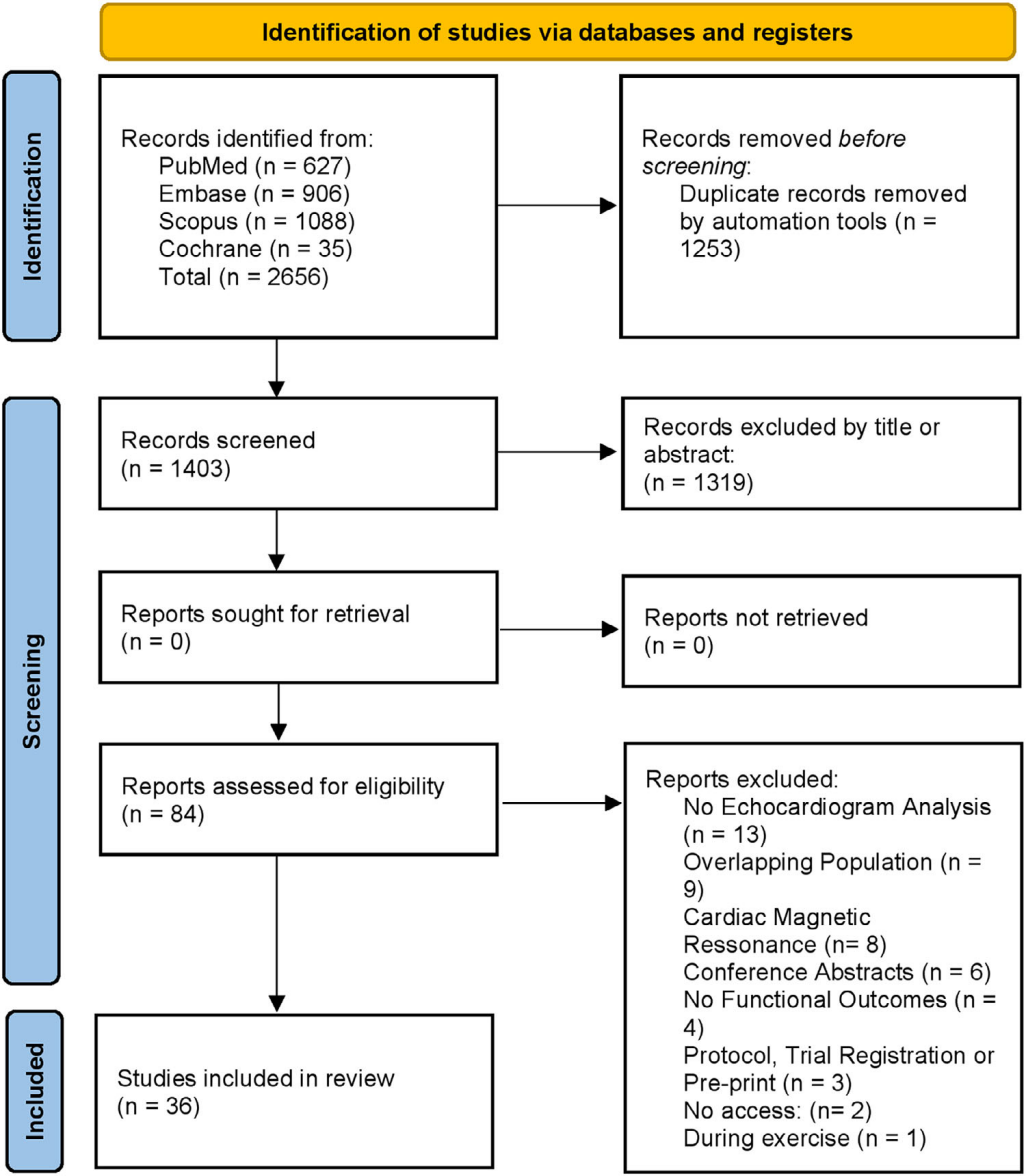
PRISMA flow diagram outlining study selection.

PRISMA process selection is available in Figure 1. The search strategy identified 2656 records, from which 1253 duplicates were removed. After screening 1403 titles and abstracts, 84 full‐text articles were reviewed, and 40 met all eligibility criteria [5, 14, 15, 16, 17, 18, 19, 20, 21, 22, 23, 24, 25, 26, 27, 28, 29, 30, 31, 32, 33, 34, 35, 36, 37, 38, 39, 40, 41, 42, 43, 44, 45, 46, 47, 48, 49, 50, 51], yielding a total of 1906 strength‐trained athletes (95% male), in 16 countries, of whom 53.8% were chronic AAS users. Across studies, exposure ranged from less than six months to more than six years. Testosterone esters were the most frequently used compounds, followed by nandrolone derivatives, trenbolone, boldenone, stanozolol, metenolone, oxandrolone, methandrostenolone, and oxymetholone. Detailed information on the reported AAS compounds, patterns of administration, dosage, and duration of use across the included studies is summarized in Supplementary Material 3. Baseline characteristics are listed in Table 1 and Supplementary Material 2. Echocardiographic outcomes in Table 2. All Supplementary Figures are in Supplementary Material 4.

**TABLE 1:** Design and characteristics of studies included in the meta‐analysis.

Study, year	Design	Country	N. AAS user / Non user	Male / Female (*N*)	Duration of use	Age—AAS / Non user (mean ± SD)
Abdullah, 2023 [14]	Cross‐sectional	Norway	101 / 71	172 / 0	11 ± 7 y	39 ± 10 / 37 ± 9
Akçakoyun, 2014 [15]	Cross‐sectional	Turkey	15 / 18	33 / 0	5.73 ± 3 y	32.5 ± 6.6 / 33.8 ± 4.1
Alizade, 2016 [16]	Cross‐sectional	Turkey	15 / 18	33 / 0	5.73 ± 3 y	32.5 ± 6.6 / 33.8 ± 4.1
Angell, 2012 [17]	Cross‐sectional	UK	28 / 19	46 / 1	> 2 y	31 ± 7 / 28 ± 8
Baggish, 2010 [5]	Cross‐sectional	USA	12 / 7	19 / 0	7.2 ± 5.6 y	39.78 ± 6.54 / 41.18 ± 6.42
Baggish, 2017 [18]	Cross‐sectional	USA	86 / 54	140 / 0	7.6 ± 5.7 y	42.67 ± 6.03 / 43.33 ± 8.38
Bigi, 2021 [19]	Cross‐sectional	Iran	18 / 35	53 / 0	> 2 y	25 ± 7.6 / 27 ± 5.7
Buhl, 2025 [20]	Cross‐sectional	Denmark	80 / 58	103 / 35	2,2 (1,2–7,2)	36 ± 9.8 / 38.9 ± 11.4
Castro, 2025 [21]	Retrospective, observational study	Brazil	14 / 8	22 / 0	>3 y	38 ± 9 / 40 ± 3
D'Andrea, 2021 [22]	Cross‐sectional	Italy	65 / 50	70 / 55	> 5 y	33.6 ± 2.5 /32.8 ± 4.4
Di Bello, 1999 [23]	Cross‐sectional	Italy	10 / 10	20 / 0	1 y	32.6 ± 5.3 / 29.5 ± 6.9
De Piccoli, 1991 [24]	Cross‐sectional	Italy	14 / 14	28 / 0	8 ± 3 w	26.1± 5.3 / 25.7±3.7
Deligiannis, 1992 [25]	Cross‐sectional	Greece	15 / 15	30 / 0	>4 y	22.8 ± 4.6 / 23.2 ± 5.2
Dickerman, 1997 [26]	Cross‐sectional	USA	8 / 8	16 / 0	6–15 y	27 ± 5.2 / 26.9 ± 7.8
Souza, 2021 [27]	Cross‐sectional	Brazil	20 / 20	40 / 0	8 ± 6 y	29 ± 5 / 29 ± 5
Fyksen, 2025 [28]	Prospective cohort	Norway	17 / 13	30 / 0	>3 y	50.3 ± 5.6 / 47.7 ± 4.9
Grandperrin, 2022 [29]	Cross‐sectional	France	20 / 15	35 / 0	> 2 y	30.8 ± 7.1 / 27.2 ± 4.5
Grandperrin, 2023 [30]	Cross‐sectional	France	24 / 20	44 / 0	> 2 y	32.3 ± 7.7 / 34.5 ± 7.7
Haji, 2022 [31]	Cross‐sectional	Iraq	10 / 20	30 / 0	> 1 y	33.6 ± 6.5 / 34.8 ± 5.1
Hajimoradi, 2012 [32]	Cross‐sectional	Iran	15 / 15	30 / 0	2.2 y	23.2 ± 3.5 / 21.2 ± 3.6
Hammoud, 2023 [33]	Cross‐sectional	Lebanon	40 / 26	66 / 0	5.80 ± 4.8 y	20 to 50 y
Hartgens, 2003 ‐ 1 [34]	Prospective Cohort	Netherlands	17 / 15	32 / 0	8–16 w	32 ± 7 / 33 ± 5
Hartgens, 2003 ‐ 2 [34]	Prospective Cohort	Netherlands	9 / 7	16 / 0	8–16 w	33 ± 9 / 31 ± 9
Ilic, 2014 [35]	Cross‐sectional	Serbia	10 / 10	20 / 0	> 3 y	27 ± 6/29 ± 6
Kahnouji, 2022 [36]	Cross‐sectional	Iran	26 / 26	52 / 0	> 4 m	24.2 ± 3.6 / 25.4 ± 4.5
Kasikcioglu, 2009 [37]	Cross‐sectional	Turkey	12 / 14	26 / 0	> 6 m	27 ± 3.46 / 26 ± 3.74
Kaya, 2019 [38]	Cross‐sectional	Turkey	89 / 92	181 / 0	> 2 y	35.1 ± 6.5 / 34.4 ± 6.9
Kouidi, 2021 [39]	Cross‐sectional	Greece	40 / 40	80 / 0	4.3 ± 0.5 y	27.4 ± 8.6 / 26.9 ± 7.8
Krieg, 2007 [40]	Cross‐sectional	Germany	14 / 11	25 / 0	8.4 ± 4.8 y	36 ± 7 / 32 ± 8
Montisci, 2010 [41]	Cross‐sectional	Italy	11 / 17	28 / 0	5.7 ± 3 y	31.2 ± 4.4 / 29.7 ± 2.9
Neto, 2016 [42]	Cross‐sectional	Brazil	15 / 15	30 / 0	3.8 ± 0.3 y	29.2 ± 4.2 / 30 ± 3.8
Nottin, 2006 [43]	Cross‐sectional	Italy	6 / 9	15 / 0	> 2 y	41 ± 6 / 38 ± 6
Palatini, 1996 [44]	Cross‐sectional	Italy	10 / 10	20 / 0	5.3 ± 3 y	28 ± 5 / 27 ± 8
Place, 2023 [45]	Cross‐sectional	UK	57 / 20	77 / 0	6.8 ± 5.1 y	30 ± 4 / 27 ± 6
Sachtleben, 1993 [46]	Cross‐sectional	USA	11 / 13	24 / 0	> 2 months (8 weeks)	26.5 ± 5.6 / 26.5 ± 5.8
Sader, 2001 [47]	Cross‐sectional	Australia	20 / 10	30 / 0	6.6 ± 1.4 y	37 ± 13.8 / 34 ± 9.4
Salke, 1985 [48]	Cross‐sectional	USA	15 / 15	30 / 0	6.7 ± 2.4 w	25.1/22.2
Thompson, 1992 [49]	Cross‐sectional	USA	12 / 11	23 / 0	3.3 ± 2.5 y	23.4 ± 3.5 / 25.8 ± 6.9
Urhausen, 2004 [51]	Cross‐sectional	Germany	17 / 15	32 / 0	8 y	30.5 ± 5 / 28 ± 4.5
Yeater, 1996 [50]	Cross‐sectional	USA	8 / 16	24 / 0	> 6 m	21 ± 2.9 / 21 ± 2.9

*Note*: All data are presented in Mean ± Standard Deviation.

Abbreviations: AAS: Androgenic Anabolic Steroids; NA: Not Available; w: weeks; m: months; N: number; y: years.

**TABLE 2:** Pooled echocardiographic outcomes by cumulative duration of AAS exposure.

ECOTT outcome	Overall analysis (Non‐stratified)	<6 months	6 months – 3 years	3 – 6 years	> 6 years	Test for subgroup differences	Meta‐regression	Figure
Measure of association	Studies (Users/control) MD (95% CI) *p*‐value | *I* ^2^	Studies (Users/control) MD (95% CI) *p*‐value | *I* ^2^	Studies (Users/control) MD (95% CI) *p*‐value | *I* ^2^	Studies (Users/control) MD (95% CI) *p*‐value | *I* ^2^	Studies (Users/control) MD (95% CI) *p*‐value | *I* ^2^	Cochrane Q test (χ^2^) df *p*‐value	*P* (slope) QM P	
E/A	29 (723/630) −0.22 (−0.30 / −0.14) *p* < 0.01 | *I* ^2^ = 85%	3 (48/52) −0.15 (−0.44/0.14) *p* = 0.32 | *I* ^2^ = 63%	9 (214 / 213) −0.29 (−0.49/−0.10) *p* < 0.01 | *I* ^2^ = 91%	9 (218 / 204) −0.09 (−0.21/0.03) *p* = 0.16 | *I* ^2^ = 73%	8 (243 / 161) −0.26 (−0.32/−0.19) *p* < 0.01 | *I* ^2^ = 20%	χ^2^ = 6.32 d*f* = 3 *p* = 0.10	*P* (slope) = 0.812 QM *p* = 0.812	F.3
E/e’	13 (579 / 457) 1.26 (0.79/1.73) *p* < 0.01 | *I* ^2^ = 83%	N/A	5 (227 / 203) 1.44 (0.33/2.64) *p* < 0.01 | *I* ^2^ = 84%	4 (160 / 134) 1.59 (0.56/2.62) *p* < 0.01 | *I* ^2^ = 90%	4 (192/120) 0.68 (0.34/1.02) *p* < 0.01 | *I* ^2^ = 0%	χ^2^ = 4.68 d*f* = 2 *p* = 0.10	*P* (slope) = 0.245 QM *p* = 0.245	F.5
LVEF	29 (835 / 740) −2.84 (−4.24/−1.44) *p* < 0.01 | *I* ^2^ = 88%	4 (62/66) −0.33 (−3.44/2.78) *p* = 0.84 | *I* ^2^ = 67.8%	9 (290 / 282) −3.07 (−5.19/−0.95) *p* < 0.01 | *I* ^2^ = 84%	11 (250 / 231) −1.39 (−3.19/0.40) *p* = 0.13 | *I* ^2^ = 81%	5 (233 / 161) −7.39 (−10.71/−4.07) *p* < 0.01 | *I* ^2^ = 54.1%	χ^2^ = 11.94 d*f* = 3 *p* < 0.01	*P* (slope) = 0.0227 QM *p* = 0.0227	F.2
GLS	13 (593 / 448) 3.27 (2.54/ 4.00) *p* < 0.01 | *I* ^2^ = 79.1%	N/A	5 (241 / 203) 2.49 (1.87/3.10) *p* < 0.01 | *I* ^2^ = 46%	3 (119 / 84) 4.60 (2.35/6.85) *p* < 0.01 | *I* ^2^ = 86%	5 (233 / 161) 3.30 (2.16/4.44) *p* < 0.01 | *I* ^2^ = 74%	χ^2^ = 4.15 d*f* = 2 *p* = 0.13	*P* (slope) = 0.343 QM *p* = 0.343	F.4
LVMI	22 (660 / 583) 18.21 (13.10/23.32) *p* < 0.01 | *I* ^2^ = 86%	3 (36 / 40) 3.33 (−12.18/18.65) *p* = 0.67| *I* ^2^ = 83%	5 (219 / 213) 18.11 (10.11/26.11) *p* < 0.01 | *I* ^2^ = 82%	9 (170 / 166) 18.96 (11.01/26.92) *p* < 0.01 | *I* ^2^ = 87%	5 (235 / 164) 26.55 (21.47/31.63) *p* < 0.01 | *I* ^2^ = 21%	χ^2^ = 10.10 d*f* = 3 *p* = 0.02	*P* (slope) = 0.00715 QM *p* = 0.00715	F.6
LVM	21 (585 / 466) 40.26 (28.79/51.73) *p* < 0.01 | *I* ^2^ = 82%	5 (73 / 77) 23.51 (−6.7/53.7) *p* = 0.13 *I* ^2^ = 89%	5 (142 / 117) 51.40 (39.80/63.00) *p* < 0.01 | *I* ^2^ = 0%	5 (117 / 102) 35.50 (12.64/58.35) *p* < 0.01 | *I* ^2^ = 89%	6 (253 / 170) 53.19 (41.08/65.29) *p* < 0.01 | *I* ^2^ = 45%	χ^2^ = 4.69 d*f* = 3 *p* = 0.20	*P* (slope) = 0.195 QM *p* = 0.195	S.F.1
IVS	34 (819 / 735) 1.17 (0.86/ 1.49) *p* < 0.01 | *I* ^2^ = 79%	6 (88 / 94) 0.82 (0.20/1.45) *p* < 0.01 | *I* ^2^ = 65%	8 (194 / 210) 0.67 (0.29/1.04) *p* < 0.01 | *I* ^2^ = 49%	12 (262 / 242) 1.41 (0.87/1.96) *p* < 0.01 | *I* ^2^ = 80%	8 (275 / 189) 1.44 (0.67/2.20) *p* < 0.01 | *I* ^2^ = 80%	χ^2^ = 6.63 d*f* = 3 *p* = 0.08	*P* (slope) = 0.0755 QM *p* = 0.0755	S.F.2
RWT	13 (453 / 382) 0.05 (0.03/0.08) *p* < 0.01 | *I* ^2^ = 92%	N/A	2 (38 / 50) 0.02 (−0.01/ 0.05) *p* = 0.14 | *I* ^2^ = 16%	8 (211 / 194) 0.06 (0.02/ 0.10) *p* < 0.01 | *I* ^2^ = 95%	3 (204 / 138) 0.05 (0.03/ 0.07) *p* < 0.01 | *I* ^2^ = 28%	χ^2^ = 3.56 d*f* = 2 *p* = 0.107	*P* (slope) = 0.601 | QM *p* = 0.601	F.7
PWT	30 (764 / 669) 0.94 (0.66/1.23) p < 0.01 | *I* ^2^ = 81%	6 (88 / 94) 0.35 (−0.12/0.81) *p* = 0.14 | *I* ^2^ = 54%	6 (161 / 160) 0.72 (0.03/1.40) *p* = 0.04 | *I* ^2^ = 90%	11 (248 / 234) 1.25 (0.74/1.75) *p* < 0.01 | *I* ^2^ = 78%	7 (267 / 181) 1.14 (0.74/1.54) *p* = 0.01 | *I* ^2^ = 49%	χ^2^ = 8.85 d*f* = 3 *p* = 0.03	*P* (slope) = 0.0306 QM *p* = 0.0306	F.8
LAVI	6 (169 / 149) 2.76 (1.57/3.95) *p* < 0.01 | *I* ^2^ = 63.5%	N/A	N/A	N/A	N/A	N/A	N/A	S.F.6
LVEDV	9 (312 / 265) 9.15 (5.34/12.96) *p* < 0.01 | *I* ^2^ = 16.71%	N/A	N/A	N/A	N/A	N/A	N/A	S.F.3
LVESV	8 (298 / 251) 9.88 (4.29/15.47) *p* < 0.01 | *I* ^2^ = 73.6%	N/A	N/A	N/A	N/A	N/A	N/A	S.F.4
E’	5 (126 / 113) −1,32 (−6.41/3.76) *p* = 0.61 | *I* ^2^ = 49%	N/A	N/A	N/A	N/A	N/A	N/A	S.F.5
PeV	6 (141 / 131) −0.97 (−3.63/1.7) *p* = 0.47 *I* ^2^ = 51.9%	N/A	N/A	N/A	N/A	N/A	N/A	S.F.7
RVGLS	3 (221 / 169) 4.36 (2.28/6.45) *p* = 0.01 *I* ^2^ = 95.3%	N/A	N/A	N/A	N/A	N/A	N/A	S.F.8
RVS	4 (112 / 97) −1.11 (−1.55/−0.68) *p* = 0.01 *I* ^2^ = 0%	N/A	N/A	N/A	N/A	N/A	N/A	S.F.9
TAPSE	4 (112/ 97) −0.99 (−3.33/1.34) *p* = 0.4 *I* ^2^ = 79.9%	N/A	N/A	N/A	N/A	N/A	N/A	S.F.10

*Notes*: All data are presented as pooled Mean Differences (MDs) with 95% Confidence Intervals (CI). Cells marked “N/A” indicate that no included study reported that outcome within the corresponding exposure‐duration stratum, reflecting a gap in the underlying primary literature rather than a data‐extraction omission by the authors; this is particularly relevant for the <6‐month GLS and E/e′ cells.

Abbreviations: AAS: Anabolic Androgenic Steroids; LVEF: Left Ventricular Ejection Fraction; GLS: Global Longitudinal Strain; E/A: Early‐to‐Late Ventricular Filling Ratio; E/e′: E‐wave to e′ Ratio; E′: Early Diastolic Mitral Annular Velocity; PeV: Peak Early Diastolic Filling Velocity; LVM: Left Ventricular Mass; LVMI: Left Ventricular Mass Index; IVS: Interventricular Septum Thickness; PWT: Posterior Wall Thickness; RWT: Relative Wall Thickness; LVEDV: Left Ventricular End‐Diastolic Volume; LVESV: Left Ventricular End‐Systolic Volume; RVGLS: Right Ventricular Global Longitudinal Strain; RVS′: Right Ventricular Systolic Velocity; TAPSE: Tricuspid Annular Plane Systolic Excursion; N/A: Not Available; S.: Supplementary; F.: Figure; *I* ^2^: Heterogeneity Index; y: Years; m: Months; QM: *Q*‐test for moderators.

### 3.2 Overall Analysis

**FIGURE 2:**
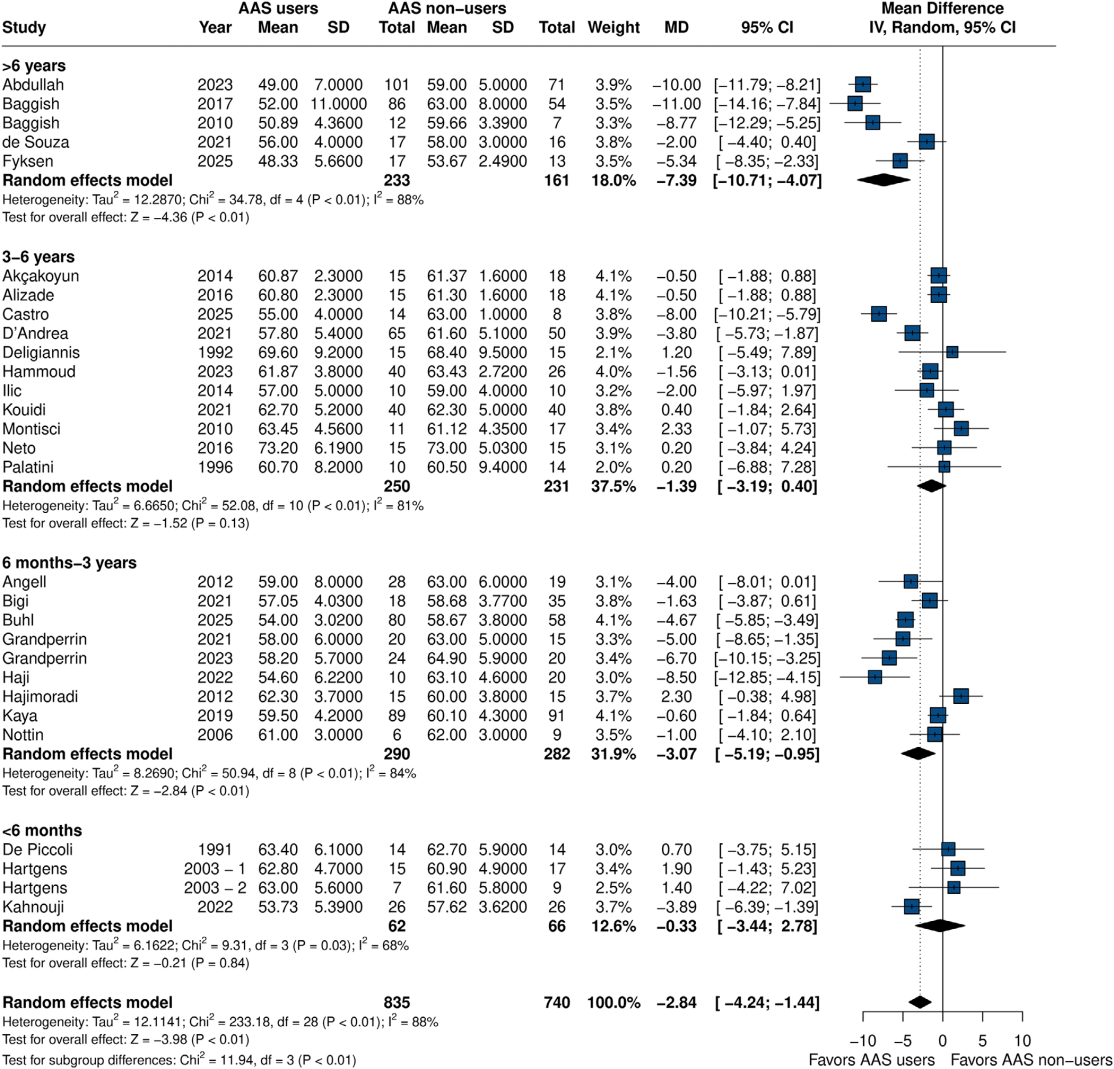
Forest plot of pooled mean differences in LVEF across cumulative durations of AAS exposure.

**FIGURE 3:**
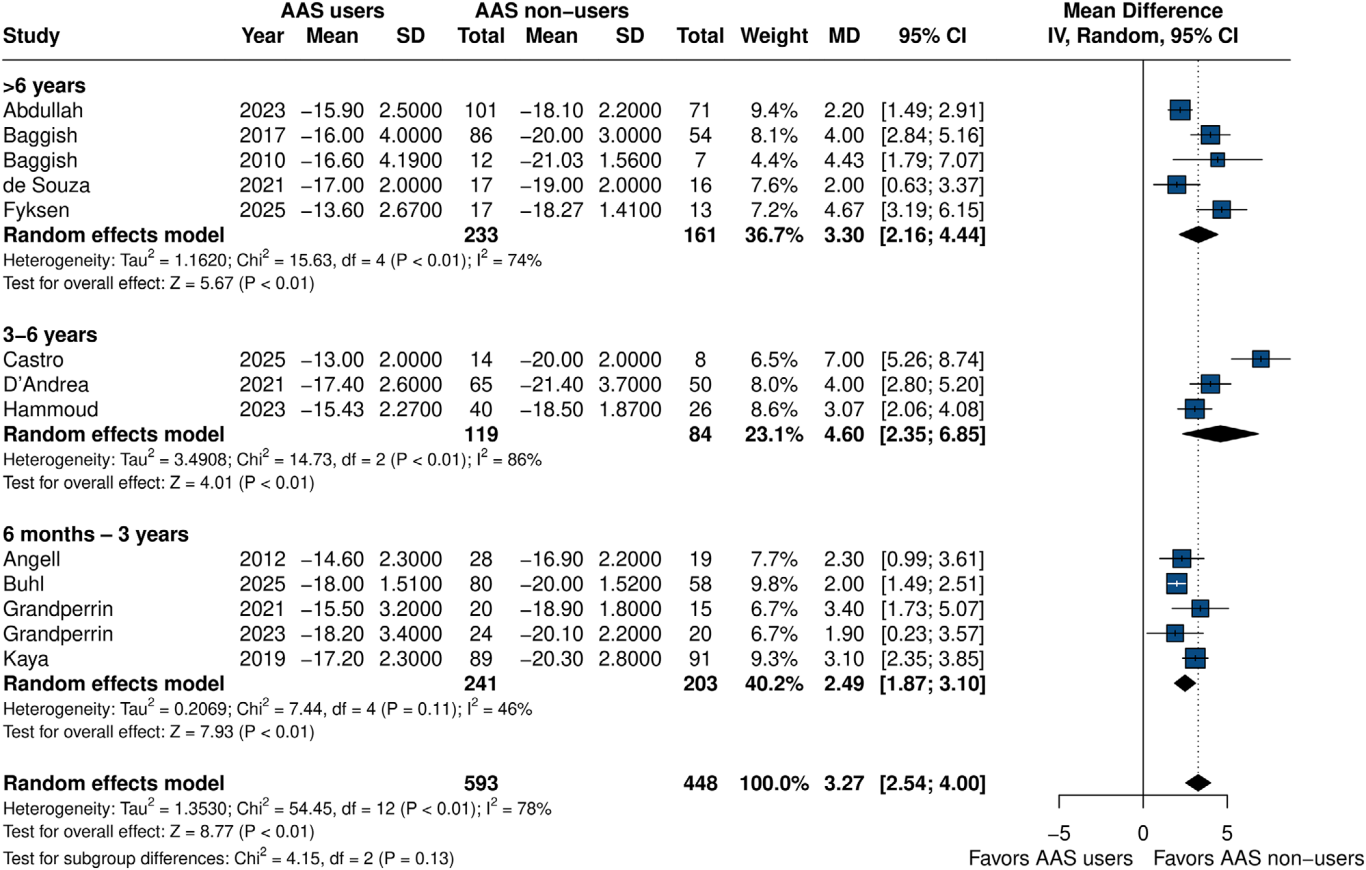
Forest plot of pooled mean differences in GLS across cumulative durations of AAS exposure.

In the non–time‐stratified analysis, LVEF was significantly reduced among AAS users compared with non‐users (MD −2.84%; 95% CI −4.24 to −1.44; *p* < 0.01; *I* ^2^ = 88%), and GLS indicated significantly impaired longitudinal strain (MD +3.27%; 95% CI 2.54 to 4.00; *p* < 0.01), consistent with subclinical systolic dysfunction detectable across both conventional and deformation‐based indices (Table 2; Figures 2 and 3).

**FIGURE 4:**
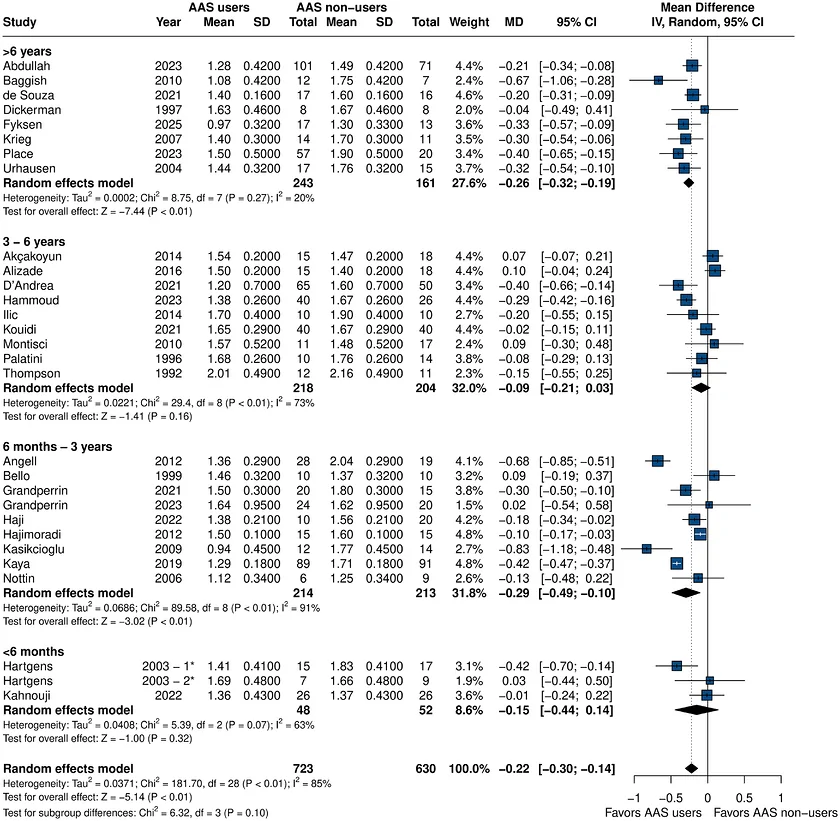
Forest plot of pooled mean differences in the E/A ratio across cumulative durations of AAS exposure.

**FIGURE 5:**
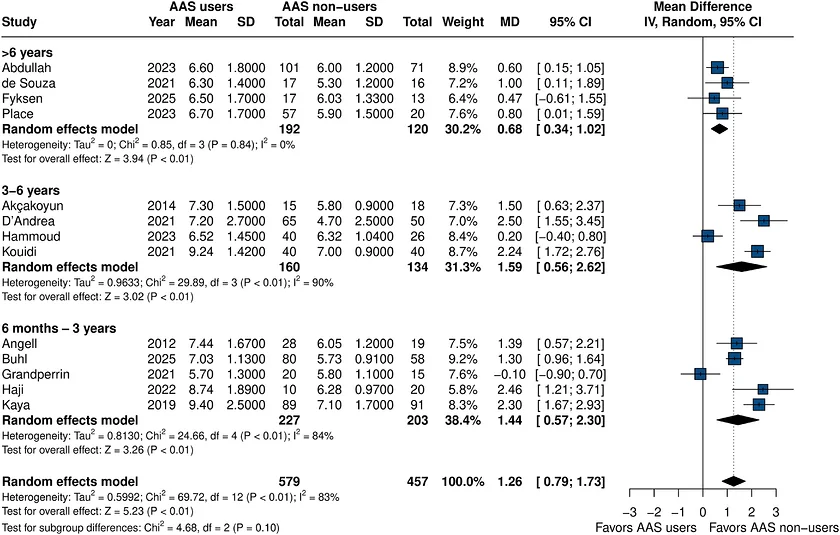
Forest plot of pooled mean differences in the E/e′ ratio across cumulative durations of AAS exposure.

Diastolic function was also altered, with a significantly reduced E/A ratio (Figure 4) and a significantly increased E/e′ ratio (Figure 5), consistent with impaired relaxation and elevated filling pressures; E′ velocity, in contrast, did not differ significantly between groups (Figure S1). LA volume index was significantly increased, supporting chronic volume overload.

**FIGURE 6:**
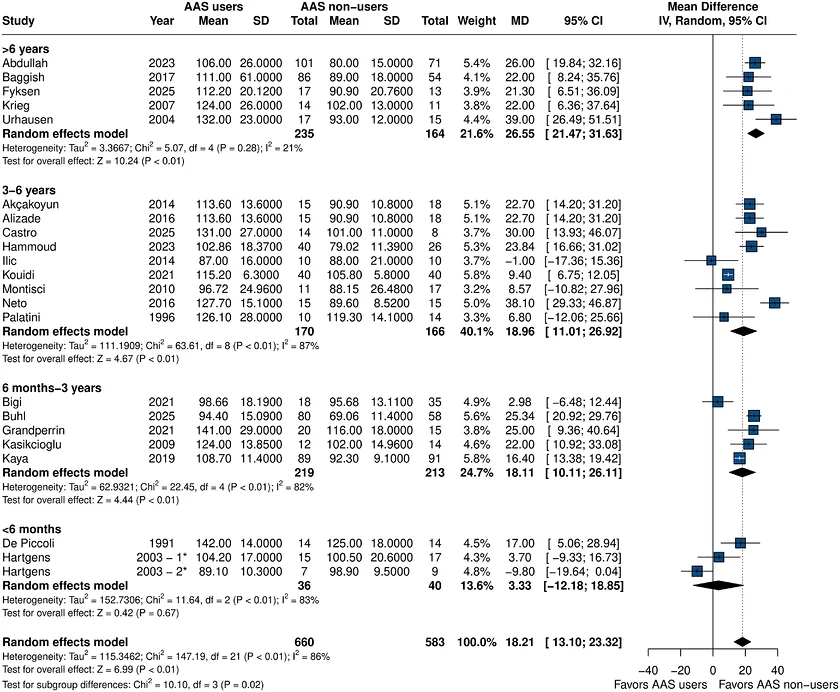
Forest plot of pooled mean differences in LVMI across cumulative durations of AAS exposure.

**FIGURE 7:**
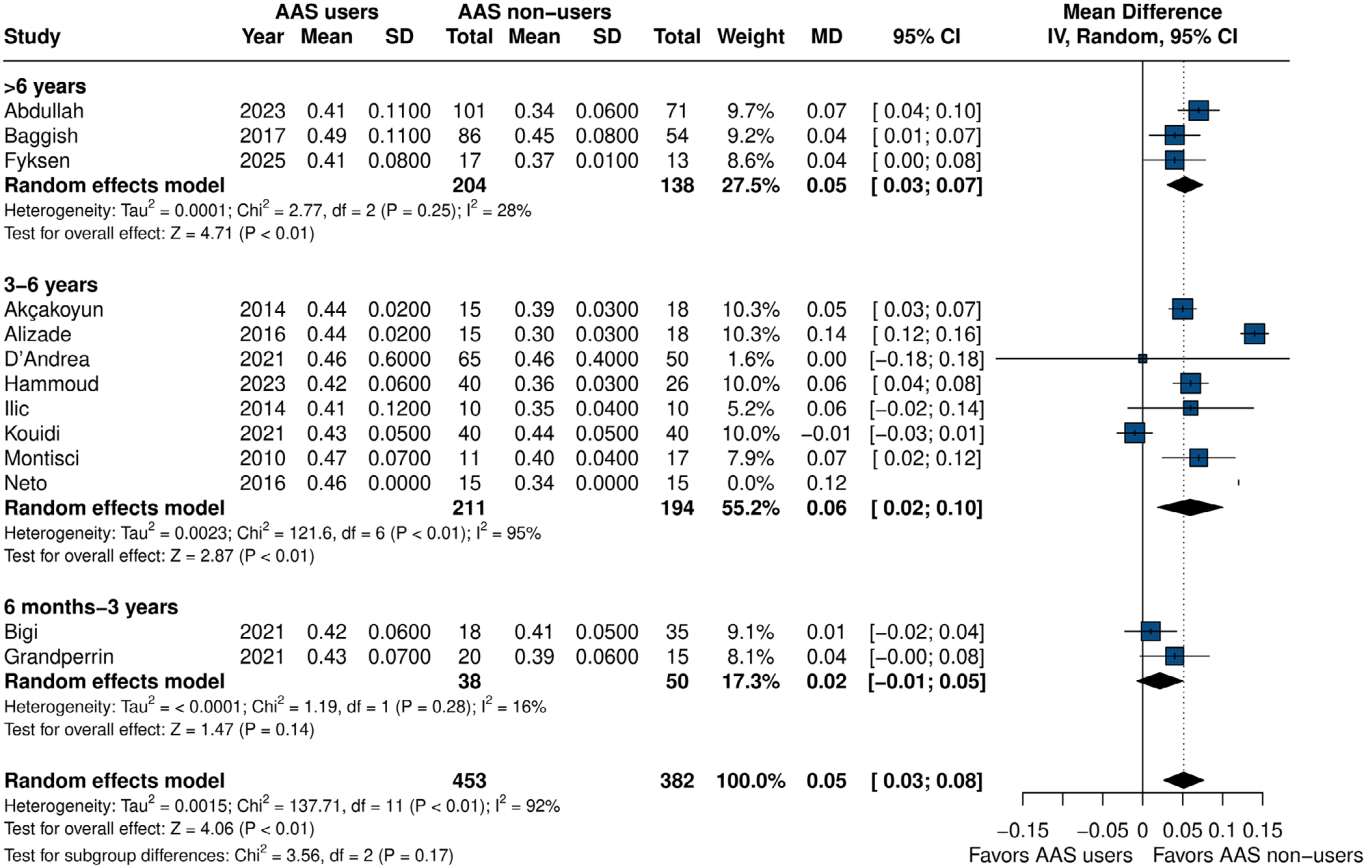
Forest plot of pooled mean differences in RWT across cumulative durations of AAS exposure.

**FIGURE 8:**
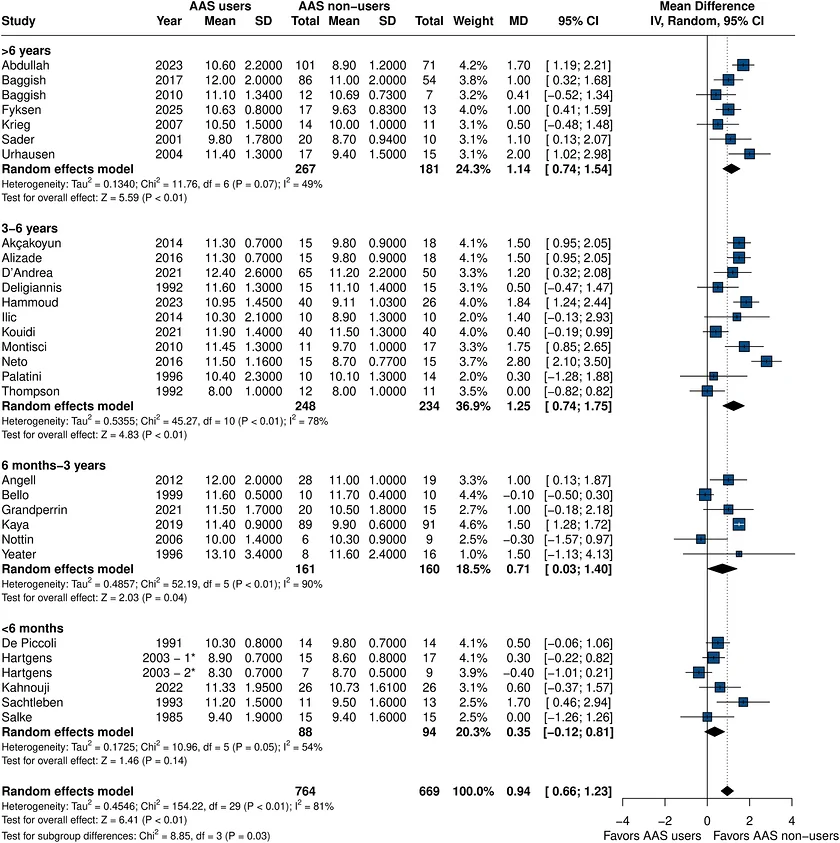
Forest plot of pooled mean differences in PWT across cumulative durations of AAS exposure.

Structural remodeling was evident across the cohort, with significant increases in LVMI, RTW, PWT, LVM, and IVS consistent with a pattern of concentric hypertrophy (Table 2; Figures 6, 7, 8, S1, and S2). The increase in LVMI was particularly pronounced (MD +18.21 g/m^2^; 95% CI 13.10 to 23.32; *p* < 0.01), with substantial between‐study heterogeneity (*I* ^2^ = 86%).

LVEDV and LVESV were both significantly increased, whereas peak early diastolic filling velocity did not differ significantly between groups (Tables 2, S3, and S4).

To explore whether these echocardiographic abnormalities varied according to the duration of AAS exposure, we subsequently stratified the analyses into four exposure categories: less than six months, six months to three years, three to six years, and more than six years.

### 3.3 Less Than Six Months of AAS Exposure

Among seven records with less than six months of AAS exposure [24, 34, 36, 37, 46, 48], structural change was already detectable in the interventricular septum, which was significantly thickened compared with non‐users. This early finding stood in contrast to the remaining parameters assessed at this stage: LVEF, LVMI, LVM, PWT, and the E/A ratio (Figures 2, 4, 6, 8, and S1) did not yet differ significantly from non‐users, and no studies in this category reported GLS, E/e′, or E′ data (Figures 3, 5, and S5). This pattern suggests that subtle structural adaptation may precede detectable systolic or diastolic impairment in the earliest phase of AAS use.

### 3.4 Six Months to Three Years of AAS Exposure

With exposure extending beyond six months but less than three years [17, 19, 20, 23, 30, 31, 32, 37, 38, 43, 50, 52], both functional and structural abnormalities became more extensive. Unlike the shorter‐exposure subgroup, LVEF and GLS were both significantly reduced (Figures 2 and 3), and diastolic function was altered, with a lower E/A ratio and higher E/e′—the first exposure category in which systolic and diastolic dysfunction were jointly evident (Figures 4 and 5). Structural remodeling also extended beyond the isolated septal thickening seen in the earlier stratum, with significant increases in LVMI, LVM, IVS, and PWT; however, RWT did not yet reach statistical significance (Table 2; Figures 6, 8, S1, and S2).

### 3.5 Three to Six Years of AAS Exposure

This pattern of structural involvement was further observed in the 3–6‐year subgroup [15, 16, 21, 22, 25, 33, 35, 39, 41, 42, 44, 49], in which LVMI, LVM, IVS, PWT, and—for the first time—RWT were all significantly increased (Figures 6, 7, 8, S1, and S2). GLS and E/e′ also remained significantly abnormal, consistent with the preceding stratum (Figures 3 and 5). Notably, however, LVEF and the E/A ratio did not differ significantly from non‐users in this exposure category, in contrast to their significant reduction at six months to three years—illustrating that not every functional parameter followed a consistent pattern across increasing exposure duration (Figures 2 and 4).

### 3.6 More Than Six Years of AAS Exposure

Among participants with more than six years of exposure [5, 14, 18, 26, 27, 28, 40, 45, 47, 51], functional impairment was again evident and reached its greatest magnitude across all strata: this subgroup showed the largest reduction in LVEF and the largest increase in LVMI observed among the four exposure categories (Figures 2 and 6), together with a return to significant reductions in the E/A ratio, alongside persistently significant GLS impairment, E/e′ elevation, and increases in IVS, RWT, and PWT (Figures 3, 4, 5, 8, and S2). Structurally, therefore, remodeling was significant across every parameter assessed in this stratum, while functionally, LVEF and the E/A ratio—attenuated in the 3–6‐year subgroup—were again significantly abnormal at longer exposure.

### 3.7 Differences Between Exposure‐Duration Strata

Across the four exposure categories, three distinct patterns emerged. IVS, GLS, and E/e′ were significantly abnormal in every stratum in which data were available, indicating a comparatively consistent abnormality across exposure duration. LVEF, LVMI, and PWT showed the clearest duration‐related pattern, with formally significant differences between exposure‐duration strata (LVEF: *p* < 0.01; LVMI: *p* = 0.02; PWT: *p* = 0.03). In contrast, the E/A ratio and LVM fluctuated between strata—significant in some categories but not others—without reaching statistical significance on formal subgroup testing (Table 2), indicating that for these two parameters a statistically confirmed duration‐response gradient was not established.

### 3.8 Right Ventricle Analysis

Although the temporal analyses above—both categorical and continuous—focused on LV structure and function, the available data also permitted an exploratory, non‐stratified assessment of selected RV parameters.

RV GLS and RV S′ were both significantly abnormal in the overall analysis, whereas TAPSE did not differ significantly between groups (Table 2; Figures S8–S10). Given the limited number of contributing studies and the absence of duration‐stratified RV data, these findings should be regarded as exploratory and complementary to the primary, LV‐focused analysis.

### 3.9 Meta‐Regression Analysis

To complement the categorical comparison of exposure‐duration strata described above, random‐effects meta‐regression was performed using cumulative AAS exposure duration as a continuous moderator, restricted to outcomes with sufficient study‐level exposure data, available in Table 2 and Supplementary Material 4.

A significant positive association with exposure duration was observed for LVMI (slope *p* = 0.00715; QM *p* = 0.00715) and PWT (slope *p* = 0.0306; QM *p* = 0.0306), indicating that pooled estimates for structural remodeling increased as a function of cumulative AAS exposure. Conversely, LVEF showed a significant negative association with exposure duration (slope *p* = 0.0227; QM *p* = 0.0227), consistent with progressively lower pooled ejection‐fraction estimates at greater cumulative exposure. This continuous‐moderator finding for LVEF was concordant with the categorical subgroup‐difference test reported above, reinforcing that the magnitude of systolic impairment differed significantly across exposure‐duration strata using both modeling approaches (Figures S11–S13).

IVS showed a positive trend that approached, but did not reach, statistical significance (slope *p* = 0.0755; QM *p* = 0.0755), consistent with the borderline result observed in the corresponding categorical subgroup comparison. No significant association with exposure duration was observed for unindexed LVM (slope *p* = 0.195; QM *p* = 0.195), the E/e′ ratio (slope *p* = 0.245; QM *p* = 0.245), GLS (slope *p* = 0.343; QM *p* = 0.343), RWT (slope *p* = 0.601; QM *p* = 0.601), or the E/A ratio (slope *p* = 0.812; QM *p* = 0.812); for these outcomes, the meta‐regression findings were concordant with the corresponding categorical subgroup‐difference tests (Table 2; Figures S14–S19).

### 3.10 Quality Assessment

The 40 studies exhibited a consistent moderate overall risk of bias. This was primarily driven by moderate risk in confounding and participant selection due to their observational design, while bias in intervention classification reflected reliance on self‐reported exposure. Risk from missing data was consistently low. Publication bias assessment via funnel plots and Egger's test showed no significant asymmetry, suggesting minimal small‐study effects despite substantial statistical heterogeneity (Supplementary Material 5).

## 4 Discussion

The main finding of this meta‐analysis is that chronic AAS use in strength‐trained athletes is associated with distinct patterns of cardiac remodeling and biventricular dysfunction that vary in magnitude across four categories of cumulative exposure duration. Compared with non‐using athletes, AAS users exhibited greater LV mass and wall thickness, larger cardiac chamber volumes, and consistent evidence of impaired myocardial deformation. Overall LVEF was also significantly reduced, an effect that was comparatively modest in the pooled, non‐stratified analysis but considerably more pronounced among users with more than six years of exposure, and for which both formal subgroup testing and continuous meta‐regression identified exposure duration as a significant moderator. RV strain was similarly impaired despite a largely preserved TAPSE. Together, these findings suggest that prolonged exposure to supraphysiological androgen levels is associated with cardiac features that may overlap with an early cardiomyopathic phenotype, with duration of use apparently relevant to at least some of the abnormalities described.

Clinical adverse effects of AAS are multisystemic and include metabolic, hematologic, hepatic, endocrine, and cardiovascular complications, ranging from subclinical LV hypertrophy to overt cardiomyopathy, arrhythmias, myocardial infarction, and sudden death [3, 53, 54, 55]. Within this spectrum, our findings highlight an important and often underrecognized stage of disease. The presence of myocardial dysfunction in otherwise asymptomatic, highly trained individuals suggests that conventional indices such as LVEF may underestimate early cardiac injury in AAS users [18, 33]. The consistent pattern of impaired relaxation, elevated filling pressures, and reduced myocardial deformation indicates that AAS‐related cardiotoxicity affects multiple domains of cardiac function and may precede overt clinical disease, reinforcing the value of deformation imaging as a sensitive tool for early detection in this population [33].

AAS encompasses a wide spectrum of synthetic testosterone derivatives with varying anabolic and androgenic potency, routes of administration, and hepatotoxicity. Commonly used agents among athletes and bodybuilders include injectable testosterone esters (e.g., testosterone enanthate, cypionate), nandrolone, trenbolone, and oral 17‐α‐alkylated compounds such as stanozolol, oxandrolone, and methandienone, often taken in high doses, combined and cycled over months to years [56, 57, 58]. Most non‐medical users are young men engaged in resistance training, bodybuilding, or power/power‐aesthetic sports; many are recreational rather than professional athletes and report appearance or physique‐driven goals (increased muscle mass, leanness, “body recomposition”) more often than competitive performance [56, 59, 60, 61].

The interaction between supraphysiological androgen exposure and the hemodynamic demands of resistance training likely underlies the maladaptive cardiac remodeling observed in AAS users. AAS activate androgen receptors in cardiomyocytes, triggering hypertrophic signaling pathways such as CaMKII‐MEF2 and mTOR, while promoting profibrotic cascades involving transforming growth factor‐beta and extracellular matrix remodeling [62, 63]. In parallel, the pressure overload associated with high‐intensity resistance exercise further amplifies these effects, shifting physiological adaptation toward pathological hypertrophy and fibrosis [64]. Additional mechanisms, including oxidative stress, endothelial dysfunction, and microvascular impairment, may contribute to diffuse myocardial injury and persistent dysfunction even after cessation [65].

Nevertheless, cardiac adaptation is strongly influenced by the type and intensity of training [66]. Resistance‐based training itself is characterized by high static load and intermittent pressure overload, which can independently promote concentric remodeling. Although traditionally described by Morganroth's hypothesis, this framework may oversimplify the heterogeneity of contemporary training practices, as the overlap between exercise‐induced remodeling and pathological phenotypes may therefore create diagnostic ambiguity in AAS users, underscoring the need for careful interpretation of imaging findings in the appropriate clinical context [67, 68].

Our structural and functional findings are consistent with this pathophysiological framework. Increased LVM, wall thickness, and LA, combined with marked impairment in LV GLS and RV strain despite relatively preserved conventional indices, suggest that chronic AAS exposure induces diffuse subclinical myocardial dysfunction. These abnormalities are best captured by deformation imaging and align with prior speckle‐tracking studies demonstrating reduced myocardial strain in steroid‐using athletes with otherwise normal conventional echocardiograms [7, 29, 33, 69].

Our findings are consistent with and extend prior observational and meta‐analytic evidence. Previous studies have demonstrated that long‐term AAS use is associated with increased LVM and impaired systolic function, with cumulative exposure emerging as a key determinant of cardiac dysfunction [7, 18]. Our meta‐regression findings corroborate this dose–duration relationship specifically for LVEF, LVMI, and PWT, while indicating that not all structural and functional parameters are equally sensitive to cumulative exposure. Prospective data, including the HAARLEM cohort, further suggest that ongoing exposure sustains or worsens these abnormalities, while only partial reverse remodeling may occur after discontinuation [70].

Building upon these observations, our analysis provides several important advances. First, by stratifying analyses into four exposure‐duration categories, we offer a more granular picture of how cardiac parameters relate to cumulative AAS exposure than has been available in prior meta‐analyses. Structural change, specifically IVS, was already detectable with less than six months of use, before any functional parameter reached significance, suggesting that morphological adaptation may be an early feature of AAS exposure. From six months onward, systolic and diastolic abnormalities became evident alongside a broader pattern of structural remodeling that persisted through the 3–6‐year stratum, even as LVEF and the E/A ratio transiently lost statistical significance in that category before both re‐emerged, more pronounced, beyond six years.

This pattern was not uniform across outcomes: formal testing for differences between strata confirmed statistically significant variation for LVEF, LVMI, and PWT, whereas the E/A ratio and unindexed LVM fluctuated between strata without reaching significance, despite numerically larger effects at longer exposure. IVS, GLS, and E/e′, by contrast, were significantly abnormal in nearly every stratum with available data, suggesting these parameters may behave as comparatively early and consistent markers of AAS‐associated cardiac involvement rather than as markers that specifically track cumulative exposure.

Our continuous meta‐regression analysis provides complementary evidence for this distinction: exposure duration was a significant moderator for LVEF, LVMI, and PWT, with IVS showing a borderline association, while no significant relationship emerged for unindexed LVM, E/e′, GLS, RWT, or the E/A ratio. We interpret the convergence of both analyses for LVEF, LVMI, and PWT as evidence that duration of exposure is relevant to at least a subset of the structural and functional abnormalities described here, while emphasizing that both approaches rest on between‐study comparisons: each exposure‐duration stratum and each meta‐regression point comprise different studies and participants rather than repeated measurements in the same individuals. These findings therefore describe a distribution of abnormalities that becomes more extensive with cumulative exposure, rather than a demonstrated within‐person progression.

Second, our findings characterize the pattern of diastolic involvement with more nuance than previously appreciated. E/e′ was significantly elevated in every stratum with available data, and LA volume was significantly increased in the overall analysis, whereas the E/A ratio reached significance in only two of the four exposure categories and E′ velocity did not differ significantly from non‐users overall. This dissociation suggests that markers of elevated filling pressure may be more consistently affected by AAS use than early relaxation velocity itself, although a single non‐significant parameter should not be taken as evidence of preserved diastolic function overall. Third, we extend the evaluation to RV mechanics in an exploratory, non‐time‐stratified analysis, showing impairment in RV strain and systolic velocity despite a largely preserved TAPSE. Collectively, these findings reinforce that AAS cardiotoxicity is not limited to concentric “athlete‐like” hypertrophy but reflects a diffuse cardiomyopathic process across the exposure spectrum [29, 52].

Our results also align with cardiac magnetic resonance studies demonstrating increased LVM, subtle reductions in biventricular function, and myocardial fibrosis in long‐term AAS users [27]. The magnitude of strain impairment observed in our analysis is consistent with these findings, supporting the role of echocardiographic GLS as a practical and sensitive front‐line marker of early AAS‐related myocardial injury in routine clinical practice.

Although most individuals included in this analysis were asymptomatic or mildly symptomatic, the more severe end of the clinical spectrum is increasingly recognized. Case series describe young AAS users presenting with dilated cardiomyopathy, malignant arrhythmias, and cardiogenic shock after prolonged exposure, in some cases requiring advanced therapies such as mechanical circulatory support or heart transplantation [71]. While the true incidence of AAS‐induced advanced heart failure in the broader AAS‐using population remains unknown, these reports underscore that what begins as “recreational” or physique‐enhancing drug use can culminate in therapies that are among the most resource‐intensive in cardiovascular medicine, with major implications for health‐care costs, lifelong follow‐up, and quality of life [72].

From a public health perspective, these findings are particularly relevant given the growing prevalence of nonmedical AAS use outside regulated competitive sports. Many users obtain these substances through informal or illicit channels without medical supervision, which may contribute to prolonged exposure and delayed recognition of cardiovascular toxicity [57, 73, 74]. This disconnect likely explains the increasing encounter of AAS‐related cardiac pathology in routine clinical practice, even among individuals with no history of participation in doping‐controlled sports [56, 59].

### 4.1 Limitations

This meta‐analysis has several limitations. First, the predominantly cross‐sectional nature of the included studies precludes definitive causal inference regarding the temporal trajectory of AAS‐induced injury; because each duration stratum comprises a distinct set of studies and participants, subgroup comparisons across strata are cross‐sectional by design and cannot, on their own, demonstrate a within‐subject temporal trajectory. Genuine confirmation of a within‐person trajectory would require longitudinal data, which we identify as a priority for future research.

Second, the heterogeneity of AAS exposure remains a significant confounder. Exposure was primarily based on self‐reporting without biochemical confirmation, and duration alone may not adequately reflect the total androgen burden, as variations in dosages and compounds were not uniformly captured. Relatedly, because most primary studies reported exposure duration as a wide mean ± SD or as an open‐ended threshold, a sensitivity analysis based on strict exclusion of studies whose exposure distribution could span more than one predefined category was not performed, as it would have removed a large, non‐random share of the evidence base; the continuous‐moderator meta‐regression and formal subgroup‐difference test described in the Statistical Analysis should be interpreted as our primary safeguards against exposure misclassification. The meta‐regression itself, however, carries its own limitations: it relies on study‐level rather than individual‐level exposure data, is therefore ecological in nature, and cannot establish that any given individual's cardiac function changes with duration of use; exposure duration may also correlate with other unmeasured study‐level characteristics, such as dose, compound, or training status, that could confound the observed associations. The number of studies contributing to individual meta‐regressions varied by outcome. It was modest for several parameters (e.g., RWT, GLS), which limits statistical power and warrants cautious interpretation of both significant and non‐significant slopes.

Third, the focus on male strength‐trained athletes limits the generalizability of our findings to women and non‐athlete users. Fourth, although our four duration‐based categories (<6 months to >6 years) were designed to balance interpretability with the granularity of available exposure data, these cut‐offs remain exploratory, and alternative categorizations might yield different results.

Fifth, several included studies had small sample sizes (*n* ≤ 10 per arm in at least five studies), which increases the influence of individual studies on stratum‐specific pooled estimates and widens confidence intervals; the continuous meta‐regression provides a complementary check on the robustness of the overall pattern that is less dependent on any single small study's influence within a discrete stratum.

Sixth, while we identified statistically significant differences in subclinical markers like GLS and E/e′, their direct clinical relevance—specifically their ability to predict hard outcomes such as heart failure or mortality in this population—remains to be established.

Seventh, RV parameters are reported only in the overall, non‐stratified analysis, given insufficient per‐stratum data for RV outcomes; these findings should be interpreted as an exploratory extension of the primary analysis rather than as evidence of a duration–response relationship specific to RV involvement.

Finally, variations in echocardiographic protocols and software vendors likely contributed to the observed statistical heterogeneity, which remained substantial (*I* ^2^ frequently exceeding 80%) even for outcomes for which exposure duration was a significant moderator; duration may therefore account for part, but clearly not all, of the between‐study variability observed across outcomes. Publication bias could not be excluded with confidence, as funnel‐plot and Egger's‐test assessments have limited power when the number of contributing studies is modest, as was the case for several outcomes in this analysis. Despite these constraints, the consistent direction of the AAS‐associated cardiac patterns across multiple parameters suggests a robust signal of cardiac involvement that warrants further longitudinal investigation.

### 4.2 Future Perspectives

Future studies should prioritize prospective longitudinal cohorts with rigorous biochemical verification of AAS exposure, standardized echocardiographic and cardiac magnetic resonance protocols (including strain and tissue characterization), and detailed assessment of drug regimens, cumulative dose, and coexisting risk factors. Because our meta‐regression relied on study‐level rather than individual‐level exposure data, individual‐patient data meta‐analysis would allow duration‐response relationships to be modeled more precisely and would help disentangle exposure duration from correlated study‐level characteristics such as dose, compound, and training status. Mechanistic investigations exploring the interplay between androgen receptor signaling, inflammation, microvascular dysfunction, and fibrosis may help identify therapeutic targets. In addition, dedicated registries of AAS‐related cardiomyopathy could clarify long‐term prognosis, reversibility, and optimal timing of advanced therapies, while RV function specifically would benefit from longitudinal, duration‐stratified assessment given the exploratory nature of the RV findings reported here. From a public health perspective, multidisciplinary collaboration between cardiology, sports medicine, psychiatry, and addiction services will be essential to develop targeted screening, harm‐reduction, and cessation strategies for high‐risk populations.

### 4.3 Conclusion

In conclusion, chronic AAS use in strength‐trained athletes is associated with LV structural remodeling and biventricular functional impairment that vary in magnitude across exposure‐duration strata. Structural change was already apparent with less than six months of use, more extensive abnormalities were observed among studies involving longer exposure, and LVEF, LVMI, and PWT showed statistically supported differences across categories on both formal subgroup testing and continuous meta‐regression, convergent evidence that duration of exposure is relevant to at least a subset of these findings.

Not every outcome followed this pattern, however: several structural and functional parameters showed no statistically confirmed duration relationship despite numerically larger effects at longer exposure, and RV abnormalities, while evident overall, rest on limited, non‐stratified data. Given the predominantly cross‐sectional, study‐level nature of the evidence base, these findings should be interpreted as a distribution of abnormalities across different groups of AAS users rather than as proof of within‐person progression.

These findings, weighed against their limitations, support a clinically meaningful association between prolonged supraphysiological androgen exposure and an early cardiomyopathic phenotype, reinforcing the importance of early cardiovascular screening, targeted counseling, and longitudinal surveillance among long‐term users.

## Funding

This research received no specific grant from any funding agency in the public, commercial, or not‐for‐profit sectors.

## Generative AI Statement

During the preparation of this work, the authors used Claude 5.0 Sonnet and Gemini to assist with the reorganization and stylistic refinement of the Graphical Abstract, Methods, Results, Discussion, and Limitations sections, to generate an initial draft of the Abstract, and to support formatting of text, tables, and the reference list. No AI tool was used for study conception, literature screening, study selection, data extraction, statistical analysis, or interpretation of results; these steps were performed exclusively by the human co‐authors. All AI‐generated suggestions were reviewed, revised, and approved by the authors, who take full responsibility for the scientific content, integrity, and accuracy of the manuscript.

## Ethics Statement

Not applicable. This study was based exclusively on previously published data. The Sírio‐Libanês Research Ethics Committee confirmed that no ethical approval was required.

## Consent

Our study did not require informed consent, given that we incorporated data from publicly available studies approved by ethics committees or institutional review boards

## Conflicts of Interest

The authors declare that they have no known competing financial or personal relationships that could have appeared to influence the work reported in this paper.

## Supporting information




**Supporting Information**: echo70611‐sup‐0001‐SuppMat.docx

**Supporting Information**: echo70611‐sup‐0002‐SuppMat.docx

**Supporting Information**: echo70611‐sup‐0003‐SuppMat.docx

**Supporting Information**: echo70611‐sup‐0004‐SuppMat.docx

**Supporting Information**: echo70611‐sup‐0005‐SuppMat.docx

## Data Availability

The data supporting the findings of this study are derived from publicly available sources. Additional details are available from the corresponding author upon reasonable request.
