# Supplementary material for: Duration‐Dependent Cardiac Effects of Anabolic‐Androgenic Steroid Use in Strength‐Trained Athletes: A Systematic Review and Meta‐Analysis
Source: Echocardiography. 2026 Aug 31;43(9):e70611. doi: 10.1111/echo.70611 (PMC13528975; doi:10.1111/echo.70611)
Supplement: Supplementary file 1 [file ECHO-43-e70611-s001.docx]

**Supplementary Material 2 – Appendix**

**Supplementary Table S1 –** Allocation and Distribution of Included Studies **Supplementary Table S2 –** Baseline Anthropometric and Hemodynamic Characteristics of the Included Populations.

**Supplementary Table S1 –** Allocation and Distribution of Included Studies

| **Study, year** | **Exposure Reported** | **Allocation Criterion** | **Final Category** |
| --- | --- | --- | --- |
| [Fyksen, 2025](https://pmc.ncbi.nlm.nih.gov/articles/PMC12323528/) | 24 (23 to 26)* | Median (IQR) | > 6 years |
| [Abdullah, 2023](https://academic.oup.com/eurjpc/article/31/5/599/7441956) | 11 ± 7 y | Mean ± SD | > 6 years |
| [Krieg, 2007](https://sci-hub.se/https://www.thieme-connect.de/products/ejournals/abstract/10.1055/s-2007-964848) | 8.4 ± 4.8 y | Mean ± SD | > 6 years |
| [Souza, 2021](https://pubmed.ncbi.nlm.nih.gov/34341973/) | 8 ± 6 y | Mean ± SD | > 6 years |
| [Urhausen, 2004](https://sci-hub.se/https://doi.org/10.1136/hrt.2003.015719) | 8 y | Reported Single Value | > 6 years |
| [Baggish, 2010](https://www.ahajournals.org/doi/10.1161/CIRCHEARTFAILURE.109.931063) | 7.2 y ± 5.6 y | Mean ± SD | > 6 years |
| [Baggish, 2017](https://www.ahajournals.org/doi/epub/10.1161/CIRCULATIONAHA.116.026945) | 7.6 ± 5.7 y | Mean ± SD | > 6 years |
| [Dickerman, 1997](https://sci-hub.se/https://doi.org/10.1097/00042752-199704000-00003) | 6 - 15 y | Range | > 6 years |
| [Place, 2023](https://echo.biomedcentral.com/articles/10.1186/s44156-023-00031-y#Tab5) | 6.8 ± 5.1 y | Mean ± SD | > 6 years |
| [Sader, 2001](https://doi.org/10.1016/S0735-1097(00)01083-4) | 6.6 ± 1.4 y | Mean ± SD | > 6 years |
| [Hammoud, 2023](https://www.jsams.org/article/S1440-2440(23)00384-5/fulltext) | 5.80 ± 4.8 y | Mean ± SD | 3 – 6 years |
| [Akçakoyun, 2014](https://doi.org/10.1155/2014/451520) | 5.73 ± 3 y | Mean ± SD | 3 – 6 years |
| [Alizade, 2016](https://sci-hub.se/https://doi.org/10.1111/echo.13243) | 5.73 ± 3 y | Mean ± SD | 3 – 6 years |
| [Montisci, 2010](https://onlinejase.com/article/S0894-7317(10)00225-7/fulltext) | 5.7 ± 3 y | Mean ± SD | 3 – 6 years |
| [Palatini, 1996](https://accp1.onlinelibrary.wiley.com/doi/epdf/10.1002/j.1552-4604.1996.tb04167.x) | 5.3 ± 3 y | Mean ± SD | 3 – 6 years |
| [D’Andrea, 2021](https://academic.oup.com/eurjpc/article/28/17/1928/6335769?login=false) | > 5 y | Reported minimum threshold (treated as point estimate) | 3 – 6 years |
| [Kouidi, 2021](https://www.mdpi.com/1660-4601/18/13/6974) | 4.3 ± 0.5 y | Mean ± SD | 3 – 6 years |
| [Deligiannis, 1992](https://onlinelibrary.wiley.com/doi/epdf/10.1111/j.1600-0838.1993.tb00358.x) | > 4 y | Reported minimum threshold (treated as point estimate) | 3 – 6 years |
| [Neto, 2016](https://sci-hub.se/https://link.springer.com/article/10.1007/s10286-017-0470-2) | 3.8 ± 0.3 y | Mean ± SD | 3 – 6 years |
| [Thompson, 1992](https://www.jacc.org/doi/epdf/10.1016/0735-1097%2892%2990478-6) | 3.3 ± 2.5 y | Mean ± SD | 3 – 6 years |
| Ilic, 2014 | > 3 y | Reported minimum threshold (treated as point estimate) | 3 – 6 years |
| [Castro, 2025](https://www.scielo.br/j/ijcs/a/g9SqLVbgHxvs6RKQ7R3TjVL/?format=pdf&lang=en) | > 3 y | Reported minimum threshold (treated as point estimate) | 3 – 6 years |
| [Buhl, 2025](https://jamanetwork.com/journals/jamanetworkopen/fullarticle/2838259) | 2.2 (1.2 - 7.2) | Median IQR | 6 months – 3 years |
| [Hajimoradi, 2012](https://pmc.ncbi.nlm.nih.gov/articles/PMC3685154/) | 2.2 y | Reported Single Value | 6 months – 3 years |
| [Grandperrin, 202](https://journals.lww.com/acsm-msse/fulltext/2022/05000/anabolic_steroids_use_is_associated_with.8.aspx)1 | > 2 y | Reported minimum threshold (treated as point estimate) | 6 months – 3 years |
| [Grandperrin, 2023](https://www.sciencedirect.com/science/article/pii/S2095254622000783?via%3Dihub) | > 2 y | Reported minimum threshold (treated as point estimate) | 6 months – 3 years |
| [Angell, 2012](https://doi.org/10.1249/mss.0b013e3182358cb0) | > 2 y | Reported minimum threshold (treated as point estimate) | 6 months – 3 years |
| [Bigi, 2021](https://sci-hub.se/https://doi.org/10.1007/s12574-020-00495-5) | > 2 y | Reported minimum threshold (treated as point estimate) | 6 months – 3 years |
| [Kaya, 2019](https://sci-hub.se/https://doi.org/10.1080/00015385.2019.1682339) | > 2 y | Reported minimum threshold (treated as point estimate) | 6 months – 3 years |
| [Nottin, 2006](https://www.ajconline.org/article/S0002-9149(05)02117-X/fulltext) | > 2 y | Reported minimum threshold (treated as point estimate) | 6 months – 3 years |
| [Haji, 2022](https://connectjournals.com/file_full_text/3541401H_653-660.pdf) | > 1 y | Reported minimum threshold (treated as point estimate) | 6 months – 3 years |
| B[ello, 1999](https://journals.lww.com/acsm-msse/fulltext/1999/04000/effects_of_anabolic_androgenic_steroids_on.4.aspx) | 1 y | Reported Single Value | 6 months – 3 years |
| [Kasikcioglu, 2009](https://www.internationaljournalofcardiology.com/article/S0167-5273(08)00010-7/fulltext) | > 6 m | Reported minimum threshold (treated as point estimate) | 6 months – 3 years |
| [Yeater, 1996](https://pmc.ncbi.nlm.nih.gov/articles/PMC1332257/?page=3) | > 6 m | Reported minimum threshold (treated as point estimate) | 6 months – 3 years |
| [Salke, 1985](https://journals.lww.com/acsm-msse/abstract/1985/12000/left_ventricular_size_and_function_in_body.14.aspx) | 6.7 ± 2.4 w | Mean ± SD | < 6 months |
| [Kahnouji, 2022](https://journal.iha.org.ir/article_158131_dd247a8009e4d4bb24aafb6bdda18196.pdf) | > 4 m | Reported Single Value | < 6 months |
| [Hartgens, 2003](https://sci-hub.se/https://www.thieme-connect.de/products/ejournals/abstract/10.1055/s-2003-40705) - 1 | 8 - 16 w | Range | < 6 months |
| Hartgens, 2003 - 2 | 8 - 16 w | Range | < 6 months |
| [De Piccoli, 1991](https://sci-hub.se/https://www.thieme-connect.de/products/ejournals/abstract/10.1055/s-2007-1024703) | 8 ± 3 w | Mean ± SD | < 6 months |
| [Sachtleben, 1993](https://journals.lww.com/acsm-msse/abstract/1993/11000/the_effects_of_anabolic_steroids_on_myocardial.6.aspx) | > 2 months (8 weeks) | Reported minimum threshold (treated as point estimate) | < 6 months |

* Accumulated Lifetime Duration of AAS-use (years)

**Supplementary Table S2 –** Baseline Anthropometric and Hemodynamic Characteristics of the Included Populations.

| **Study, year** | **N. AAS user / Non user** | **BMI - AAS / Non User** | **HR - AAS user / Non user** | **SBP - AAS user/ Non user** | **Diastolic Blood Pressure - AAS user / N user** |
| --- | --- | --- | --- | --- | --- |
| [Abdullah, 2023](https://academic.oup.com/eurjpc/article/31/5/599/7441956) | 101 / 71 | 30.0 ± 4.2 / 28.2 ± 3.1 | 72 ± 13 / 66 ± 13 | 141 ± 17 / 133 ± 11 | 77 ± 10 / 74 ± 11 |
| [Akçakoyun, 2014](https://doi.org/10.1155/2014/451520) | 15 / 18 | 29.1 ± 4.4 / 26.3 ± 3.2 | 72.2 ± 13.4 / 68.7± 10.4 | 118.51 ± 9.8 / 120 ± 13.3 | 78.51 ± 6.9 / 80.37 ± 6.49 |
| [Alizade, 2016](https://sci-hub.se/https://doi.org/10.1111/echo.13243) | 15 / 18 | 29.1 ± 4.4 / 26.3 ± 3.2 | 72.2 ± 13.4 // 68.74 ± 10.4 | 118.51 ± 9.8 / 120 ± 13.3 | 78.51 ± 6.9 / 80.37 ± 6.49 |
| [Angell, 2012](https://doi.org/10.1249/mss.0b013e3182358cb0) | 28 / 19 | 30.23 ± 4.23 / 25.32 ± 2.92 | 79 ± 12 / 64 ± 13 | 133 ± 15 / 126 ± 12 | 67 ± 7 / 73 ± 10 |
| [Baggish, 2010](https://www.ahajournals.org/doi/10.1161/CIRCHEARTFAILURE.109.931063) | 12 / 7 | 30.3 ± 3.01 / 29.11 ± 4.50 | NA | NA | NA |
| [Baggish, 2017](https://www.ahajournals.org/doi/epub/10.1161/CIRCULATIONAHA.116.026945) | 86 / 54 | 31 ± 3 / 43.33 ± 8 | 65 ± 5 / 62 ± 6 | 118 ± 11 / 115 ± 10 | 76 ± 9 / 72 ± 9 |
| [Bigi, 2021](https://sci-hub.se/https://doi.org/10.1007/s12574-020-00495-5) | 18 / 35 | NA | NA | 136 ± 12.2 / 138 ±11.8 | 84.4 ± 8.6 / 82.1 ± 7.3 |
| [Buhl, 2025](https://jamanetwork.com/journals/jamanetworkopen/fullarticle/2838259) | 80 / 58 | 25.5 ± 34 / 28.9 ± 3.7 | NA | 132 ± 13.5 / 132 ± 10.6 | 77 ± 9.8 / 76 ± 11.4 |
| [Castro, 2025](https://www.scielo.br/j/ijcs/a/g9SqLVbgHxvs6RKQ7R3TjVL/?format=pdf&lang=en) | 14 / 8 | NA | NA | NA | NA |
| [D’Andrea, 2021](https://academic.oup.com/eurjpc/article/28/17/1928/6335769?login=false) | 65 / 50 | NA | 68.4 ± 8.8 / 67.4 ± 7.7 | 139.8 ± 8.5 / 128.4 ± 7.3 | 85.3 ± 5.5 / 81.3 ± 4.7 |
| Di B[ello, 1999](https://journals.lww.com/acsm-msse/fulltext/1999/04000/effects_of_anabolic_androgenic_steroids_on.4.aspx) | 10 / 10 | 49.1 ± 2.9 / 46.3 ± 2.7 | 71.4 ± 16.6 / 77.7 ± 15.4 | 135 ± 19.3 / 137.5 ± 7.9 | 89 ± 11.9 / 87 ± 7.8 |
| [De Piccoli, 1991](https://sci-hub.se/https://www.thieme-connect.de/products/ejournals/abstract/10.1055/s-2007-1024703) | 14 / 14 | NA | 65.3 ± 15.8 / 64.2 ± 8.2 | 142.2 ± 10.9 / 136.1 ± 12.2 | 83.3 ± 5.0 / 87.2 ± 8.7 |
| [Deligiannis, 1992](https://onlinelibrary.wiley.com/doi/epdf/10.1111/j.1600-0838.1993.tb00358.x) | 15 / 15 | NA | 71.6 ± 2.8 / 70.2 ± 3.1 | 145.1 ± 9.6 / 125.8 ± 10.4 | 83.4 ± 4.6 / 79.8 ± 5.1 |
| [Dickerman, 1997](https://sci-hub.se/https://doi.org/10.1097/00042752-199704000-00003) | 8 / 8 | 34.1 ± 2.8 / 32± 4.3 | 64.5 ± 7.3 / 69.2 ± 6.6 | NA | NA |
| [Souza, 2021](https://pubmed.ncbi.nlm.nih.gov/34341973/) | 20 / 20 | 31 ± 3 / 25 ± 2 | NA | NA | NA |
| [Fyksen, 2025](https://pmc.ncbi.nlm.nih.gov/articles/PMC12323528/) | 17 / 13 | 31.1 ± 4.2 / 30.4 ± 7.3 | 64.1 ± 11.3 / 61.8 ± 8.3 | 133.6 ± 21.8 / 121 ± 8.3 | 82.8 ± 14.5 / 72.9 ±9.9 |
| [Grandperrin, 202](https://journals.lww.com/acsm-msse/fulltext/2022/05000/anabolic_steroids_use_is_associated_with.8.aspx)2 | 20 / 15 | NA | 68 ± 10 / 67 ± 14 | 125 ± 8 / 130 ± 11 | 76 ± 7 / 71 ± 8 |
| [Grandperrin, 2023](https://www.sciencedirect.com/science/article/pii/S2095254622000783?via%3Dihub) | 24 / 20 | NA | 68 ± 11 / 62 ± 13 | 127 ± 8 / 129 ± 10 | 77 ± 8 / 71 ± 9 |
| [Haji, 2022](https://connectjournals.com/file_full_text/3541401H_653-660.pdf) | 10 / 20 | 28.1 ± 4 / 25.75 ± 2.8 | 63.7 ± .6 / 76.2 ± 6.4 | 127 ± 5.3 / 123 ± 5.4 | 83.5 ± 6.6 / 82.2 ± 5.5 |
| [Hajimoradi, 2012](https://pmc.ncbi.nlm.nih.gov/articles/PMC3685154/) | 15 / 15 | 27.1 ± 1.8 / 24.7 ± 2.1 | NA | NA | NA |
| [Hammoud, 2023](https://www.jsams.org/article/S1440-2440(23)00384-5/fulltext) | 40 / 26 | 29.1 ± 3 / 26.1 ± 2.9 | 76.3 ± 10.2 / 72 ± 14 | 127.9 ± 11 / 120.6 ± 13.4 | 75.4 ± 10 / 73.3 ± 8.6 |
| [Hartgens, 2003](https://sci-hub.se/https://www.thieme-connect.de/products/ejournals/abstract/10.1055/s-2003-40705) - 1 | 17 / 15 | NA | NA | 131 ± 12 / 129 ±14 | 83 ± 6 / 81 ± 9 |
| Hartgens, 2003 - 2 | 9 / 7 | NA | NA | 133 ± 16 / 130 ± 14 | 83 ± 13 / 84 ± 6 |
| Ilié, 2014 | 10 / 10 | NA | 77 ± 16 / 68 ± 15 | 133 ± 23/128 ± 15 | 88 ± 14 / 80 ± 11 |
| [Kahnouji, 2022](https://journal.iha.org.ir/article_158131_dd247a8009e4d4bb24aafb6bdda18196.pdf) | 26 / 26 | 26.3±1.4 / 25.6±2.8 | NA | NA | NA |
| [Kasikcioglu, 2009](https://www.internationaljournalofcardiology.com/article/S0167-5273(08)00010-7/fulltext) | 12 / 14 | NA | 60 ± 6.92 / 55 ± 7.48 | 120 ± 10.39 / 112 ± 11.22 | 79 ± 6.92 / 73 ± 7.48 |
| [Kaya, 2019](https://sci-hub.se/https://doi.org/10.1080/00015385.2019.1682339) | 89 / 92 | 29.3 ± 3.9 / 29.1 ± 4.1 | 71.6 ± 7.6 / 70.4 ± 7.2 | 126.8 ±12.2/124.5±10.4 | 83.8 ± 5.7/83.1 ± 5.2 |
| [Kouidi, 2021](https://www.mdpi.com/1660-4601/18/13/6974) | 40 / 40 | 26.6 ± 2.6 / 26.2 ± 2.3 | NA | NA | NA |
| [Krieg, 2007](https://sci-hub.se/https://www.thieme-connect.de/products/ejournals/abstract/10.1055/s-2007-964848) | 14 / 11 | 31 ± 3 / 26 ± 2 | NA | 135 ± 10 / 130 ± 5 | 85 ± 5 / 80 ± 10 |
| [Montisci, 2010](https://onlinejase.com/article/S0894-7317(10)00225-7/fulltext) | 11 / 17 | 29.2 ± 2.2 / 25.4 ± 2.7 | 70.7 ± 8.2 / 62 ± 9 | 134.5 ± 15.7 / 129.3 ± 15.7 | 84.5 ± 9.3 / 84.3 ± 10.1 |
| [Neto, 2016](https://sci-hub.se/https://link.springer.com/article/10.1007/s10286-017-0470-2) | 15 / 15 | 30.8 ± 1.5 / 27.7 ± 3 | 69.4 ± 2.9 / 68.9 ± 1.6 | NA | NA |
| [Nottin, 2006](https://www.ajconline.org/article/S0002-9149(05)02117-X/fulltext) | 6 / 9 | NA | 72 ± 6 / 68 ± 14 | 132 ± 10 / 122 ± 11 | 87 ± 8 / 77 ± 12 |
| [Palatini, 1996](https://accp1.onlinelibrary.wiley.com/doi/epdf/10.1002/j.1552-4604.1996.tb04167.x) | 10 / 10 | 29.4 ± 3.0 / 25.7 ± 1.3 | 74 ± 10 / 68 ± 7 | 124 ± 14 / 128 ± 11 | 80 ± 14 / 74 ± 7 |
| [Place, 2023](https://echo.biomedcentral.com/articles/10.1186/s44156-023-00031-y#Tab5) | 57 / 20 | NA | 68 ± 11 / 56 ± 9 | 127 ± 10 / 119 ± 11 | 74 ± 9 / 72 ± 10 |
| [Sachtleben, 1993](https://journals.lww.com/acsm-msse/abstract/1993/11000/the_effects_of_anabolic_steroids_on_myocardial.6.aspx) | 11 / 13 | NA | NA | NA | NA |
| [Sader, 2001](https://doi.org/10.1016/S0735-1097(00)01083-4) | 20 / 10 | 29 ± 7.1 / 26 ± 2.5 | NA | 127 ± 12 / 119 ± 12 | 74 ± 21.9 / 71 ± 17 |
| [Salke, 1985](https://journals.lww.com/acsm-msse/abstract/1985/12000/left_ventricular_size_and_function_in_body.14.aspx) | 15 / 15 | NA | NA | NA | NA |
| [Thompson, 1992](https://www.jacc.org/doi/epdf/10.1016/0735-1097%2892%2990478-6) | 12 / 11 | NA | 82 ± 16 / 80.7 ± 14.3 | 122.8 ± 9.9 / 124.5 ± 17.3 | 78.0 ± 12 / 80.4 ± 8.4 |
| [Urhausen, 2004](https://sci-hub.se/https://doi.org/10.1136/hrt.2003.015719) | 17 / 15 | NA | 73 ± 12 / 71 ± 11 | 140 ± 10 / 125 ± 10 | 85 ± 10 / 80 ± 10 |
| [Yeater, 1996](https://pmc.ncbi.nlm.nih.gov/articles/PMC1332257/?page=3) | 8 / 16 | 31 ± 3 / 28 ± 3 | 79 ± 11 / 64 ± 10 | NA | NA |

All data are presented in Mean ± Standard Deviation; AAS: Androgenic Anabolic Steroids; BMI: Body Mass Index; DBP: Diastolic Blood Pressure; HR: Heart Rate; NA: Not Available; SBP: Systolic Blood Pressure; W: weeks; M: months; Y: Years; N: Number.
