# Supplementary material for: Duration‐Dependent Cardiac Effects of Anabolic‐Androgenic Steroid Use in Strength‐Trained Athletes: A Systematic Review and Meta‐Analysis
Source: Echocardiography. 2026 Aug 31;43(9):e70611. doi: 10.1111/echo.70611 (PMC13528975; doi:10.1111/echo.70611)
Supplement: Supplementary file 2 [file ECHO-43-e70611-s002.docx]

**Supplementary Material 4 – Appendix**

**Supplementary Figure S1 –** Left Ventricle Mass (LVM)

**Supplementary Figure S2 –** Interventricular Septum Thickness (IVS)

**Supplementary Figure S3 –** Left ventricular end-diastolic volume (LVEDV)

**Supplementary Figure S4 –** Left ventricular end-systolic volume (LVESV)

**Supplementary Figure S5 –** Mitral annular early diastolic tissue Doppler velocity (e’)

**Supplementary Figure S6 –** Left Atrium Volume Index (LAVI)

**Supplementary Figure S7 –** Peak early diastolic velocity (PeV)

**Supplementary Figure S8 –** Right Ventricular Global Longitudinal Strain (RVGLS)

**Supplementary Figure S9 –** Right ventricular S′ velocity (RV S’)

**Supplementary Figure S10 –** Tricuspid annular plane systolic excursion (TAPSE)

**Supplementary Figure S11 –** LVEF meta-regression

**Supplementary Figure S12 –** LVMI meta-regression

**Supplementary Figure S13 –** PWT meta-regression

**Supplementary Figure S14 –** IVS meta-regression

**Supplementary Figure S15 –** LVM meta-regression

**Supplementary Figure S16 –** E/e’ meta-regression

**Supplementary Figure S17 –** RWT meta-regression

**Supplementary Figure S18 –** GLS meta-regression

**Supplementary Figure S19 –** E/A meta-regression

**Supplementary Figure S1 –** Left Ventricle Mass (LVM)

**Supplementary Figure S2 –** Interventricular Septum Thickness (IVS)

**Supplementary Figure S3 –** Left ventricular end-diastolic volume (LVEDV)

**Supplementary Figure S4 –** Left ventricular end-systolic volume (LVESV)

**Supplementary Figure S5 –** Mitral annular early diastolic tissue Doppler velocity (e’)

**Supplementary Figure S6 –** Left Atrium Volume Index (LAVI)

**Supplementary Figure S7 –** Peak early diastolic velocity (PeV)

**Supplementary Figure S8 –** Right Ventricular Global Longitudinal Strain (RVGLS)

**Supplementary Figure S9 –** Right ventricular S′ velocity (RV S’)

**Supplementary Figure S10 –** Tricuspid annular plane systolic excursion (TAPSE)

**Supplementary Figure S11 –** LVEF meta-regression

**Supplementary Figure S12 –** LVMI meta-regression

**Supplementary Figure S13 –** PWT meta-regression

**Supplementary Figure S14 –** IVS meta-regression

**Supplementary Figure S15 –** LVM meta-regression

**Supplementary Figure S16 –** E/e’ meta-regression

**Supplementary Figure S17 –** RWT meta-regression

**Supplementary Figure S18 –** GLS meta-regression

**Supplementary Figure S19 –** E/A meta-regression
