# Supplementary material for: Duration‐Dependent Cardiac Effects of Anabolic‐Androgenic Steroid Use in Strength‐Trained Athletes: A Systematic Review and Meta‐Analysis
Source: Echocardiography. 2026 Aug 31;43(9):e70611. doi: 10.1111/echo.70611 (PMC13528975; doi:10.1111/echo.70611)
Supplement: Supplementary file 3 [file ECHO-43-e70611-s003.docx]

Supplementary Material 3: Reported patterns of anabolic-androgenic steroid (AAS) use, dosage, and duration in the included studies.

| **Study, Year** | **AAS Use Description (as reported by the authors)** | **Type of AAS (Number of Users, if reported)** | **AAS (Administration Pattern)** | **Mean Weekly Dose (mg·wk⁻¹, mean ± SD)** | **Duration of AAS Use (years, mean ± SD)** | **Dose Range (mg·wk⁻¹)** |
| --- | --- | --- | --- | --- | --- | --- |
| Hammoud, 2023 | “Intramuscular injections of testosterone esters (cypionate, propionate, enanthate), boldenone undecylenate, trenbolone (acetate, enanthate), drostanolone (propionate, enanthate), methenolone enanthate, nandrolone decanoate, and chlorodehydromethyl-testosterone (turinabol). In addition, oral AASs were taken as oxandrolone, stanozolol, and oxymetholone.” | N/A | N/A | N/A | N/A | N/A |
| [Grandperrin, 2023](https://www.sciencedirect.com/science/article/pii/S2095254622000783?via%3Dihub) |  | Testosterone (21) Nandrolone (17) Decabolone (15) Trenbolone (13) Masterone (7) Boldenone (5) | Testosterone (IM) Nandrolone (IM) Decabolone (IM) Trenbolone (IM) Masterone (IM) Boldenone (IM)  (100 % IM) | 588 ± 225 mg  450 ± 140 mg 340 ± 70 mg 425 ± 35 mg 475 ± 50 mg 400 ± 50 mg | 8.8 ± 2.4 5.1 ± 1.1 4.5 ± 1.5 6.9 ± 1.5 4.5 ± 1.5  5.8 ± 1.4 |  |
| [Neto, 2016](https://sci-hub.se/https:/link.springer.com/article/10.1007/s10286-017-0470-2) | “Nandrolone decanoate (80%), testosterone propionate (73%), stanozolol (57%), and testosterone cypionate (31%).” |  |  |  |  |  |
| [Sader, 2001](https://doi.org/10.1016/S0735-1097(00)01083-4) | “Stanozolol (70%) and nandrolone (50%)” |  |  |  |  |  |
| [Grandperrin, 2021](https://journals.lww.com/acsm-msse/fulltext/2022/05000/anabolic_steroids_use_is_associated_with.8.aspx) |  | Testosterone (17) Nandrolone (14) Decabolone (14) Trenbolone (10) Masterone (5) Boldenone (3) | Testosterone (IM) Nandrolone (IM) Decabolone (IM) Trenbolone (IM) Masterone (IM) Boldenone (IM)  (100% IM) | 607 ± 247 mg 475 ± 150 mg 350 ± 75 mg 450 ± 25 mg 450 ± 50 mg 450 ± 50 mg | 9.0 ± 2.5 5.0 ± 1.0 5.0 ± 1.5 7.0 ± 1.5 5.0 ± 1.0 6.0 ± 1.5 | 400–1000 350–600 250–500 400–500 300–500 300–500 |
| [Akçakoyun, 2014](https://doi.org/10.1155/2014/451520) | “The orally self-administered drugs were oxymetholone and stanozolol, and the injectable steroids were nandrolone, stanozolol, and testosterone propionate.“ | N/A | N/A | N/A | N/A | N/A |
| [SACHTLEBEN, 1993](https://journals.lww.com/acsm-msse/abstract/1993/11000/the_effects_of_anabolic_steroids_on_myocardial.6.aspx) | “Most frequently used steroids were Dianabol (methanandrostenolone), Anadrol-50 (oxymetholone), and Deca-derabolin (nandrolone decanoate).” |  | Methandrostenolone (Oral) Oxandrolone (Oral) Stanozolol (Oral) Oxymetholone (Oral) Methyltestosterone (Oral) Fluoxymesterone (Oral) Methenolone acetate (Oral) Nandrolone decanoate (IM) Testosterone cypionate (IM) Testosterone enanthate (IM) Testosterone propionate (IM) Bolasterone (IM) Boldenone undecylenate (IM) Methenolone enanthate (IM) | N/A | N/A | N/A |
| [Kahnouji, 2022](https://journal.iha.org.ir/article_158131_dd247a8009e4d4bb24aafb6bdda18196.pdf) | “AAS abuse for at least 16 weeks of testosterone or nandrolone injection.” | N/A | N/A | N/A | N/A | N/A |
| [Place, 2023](https://echo.biomedcentral.com/articles/10.1186/s44156-023-00031-y#Tab5) |  | Testosterone 400 (31) Testosterone enanthate 300 (45) Human growth hormone (12) Boldenone (3) Yohimbine (1) Nandrolone decanoate (1) | Testosterone 400 (IM/Oral tablet) Testosterone enanthate 300 (IM/Oral tablet) Human growth hormone (IM) Boldenone (IM/Oral tablet) Yohimbine (N/A) Nandrolone decanoate (N/A)  (81% injection, 19% oral tablet) | N/A | N/A | N/A |
| [Kasikcioglu, 2009](https://www.internationaljournalofcardiology.com/article/S0167-5273(08)00010-7/fulltext) | “The AAS were being taken orally in various combinations and different cycles and included: stanozolol, metenolone, nandrolone, testosterone, oxymetholone, methandrostenolone, and drostanolone.“ | N/A | N/A | N/A | N/A | N/A |
| [**Deligiannis, 1992**](https://onlinelibrary.wiley.com/doi/epdf/10.1111/j.1600-0838.1993.tb00358.x) | “Almost all these athletes used oral methandrostenolone or stanolone (more than 20 mg total daily dose) and also injectable steroids, such as nandrolone decanoate (more than 200 mg weekly). “ |  |  |  |  |  |
| [**Bigi, 2021**](https://sci-hub.se/https:/doi.org/10.1007/s12574-020-00495-5) | “Oxymetholone 25 mg/day for at least 2 years” | N/A | N/A | N/A | N/A | N/A |
| [Alizade, 2016](https://sci-hub.se/https:/doi.org/10.1111/echo.13243) | “The orally self-administered drugs were oxymetholone and stanozolol, and the injectable steroids were nandrolone, stanozolol, and testosterone propionate.” | N/A | N/A | N/A | N/A | N/A |
| [Ilic, 2014](https://doiserbia.nb.rs/img/doi/0042-8450/2014/0042-84501300046I.pdf) | “All the subjects used the combination of oral and injectable substances (methandienone, stanozolol, nandrolone decanoate, and testosterone).” | N/A | N/A | N/A | N/A | N/A |
| [**Hajimoradi, 2012**](https://pmc.ncbi.nlm.nih.gov/articles/PMC3685154/) | “Chronic anabolic steroid abuse for the preceding two years and at least twice weekly or eight times per month of testosterone or nandrolone injection or daily oxymetholone ingestion” | N/A | N/A | N/A | N/A | N/A |
| [Francis Ribeiro de Souza, 2021](https://pubmed.ncbi.nlm.nih.gov/34341973/) |  | Testosterone (17) Nandrolone (4) Boldenone (15) Trenbolone (9) Stanozolol (13) |  | 575 ± 220 mg 630 ± 125 mg 500 ± 100 mg 480 ± 200 mg 350 ± 180 mg |  |  |
| [**Bello, 1999**](https://journals.lww.com/acsm-msse/fulltext/1999/04000/effects_of_anabolic_androgenic_steroids_on.4.aspx) |  | Testosterone proprionate Testosterone cypionate  Oxandrolone  Stanozolol  Methenolone enanthate | N/A | N/A | N/A | 300 - 500 200 - 350 100 -150 75 -100 300 - 600 |
| [**Palatini, 1996**](https://accp1.onlinelibrary.wiley.com/doi/epdf/10.1002/j.1552-4604.1996.tb04167.x) |  | Methenolone enantate (6) Oxandrolone (6) Stanozolol (6) Nandrolone decanoate (4) Nandrolone undecylenate (4) Testosterone propionate (4) Stanozolol (4) Testosterone enantate and propionate (3) | Methenolone enantate (Intramuscular) Oxandrolone (Oral) Stanozolol (Oral) Nandrolone decanoate (Intramuscular) Nandrolone undecylenate (Intramuscular) Testosterone propionate (Intramuscular) Stanozolol (Oral) Testosterone enantate and propionate (Intramuscular)  (62,5% IM \| 37,5% Oral) | 133,3 mg 94,2 mg 65,0 mg 117,5 mg 130,0 mg 100,0 mg 92,5 mg 573,3 mg |  | 50 - 300 85 - 105 50 - 70 50 - 150 100 - 200 50 - 150 50 - 150 100 - 1500 |
| [**Thompson, 1992**](https://www.jacc.org/doi/epdf/10.1016/0735-1097%2892%2990478-6) |  | Testosterone (2) Testosterone cypionate (3) Testosterone propionate (3) Boldenone (1) Nandrolone decanoate (5) Oxandrolone (3) Trenbolone (1) Norethandrolone (1)  Oxymetholone (2) Stanozolol (3) Drostanolone (3) Dianabol (1) Methyltestosterone (1) | N/A | N/A | N/A | N/A |
| [D’Andrea, 2021](https://academic.oup.com/eurjpc/article/28/17/1928/6335769?login=false) | “Intramuscular injections were metenolone, nandrolone, and esters of testosterone. The steroids taken orally included fluoxymesterone, mesterolone, metenolone, metandienone, oxandrolone, and oxymetholone.” | N/A | N/A | N/A | N/A | N/A |
| [Urhausen, 2004](https://sci-hub.se/https:/doi.org/10.1136/hrt.2003.015719) | “Intramuscular injections were boldenone, drostanolone, formebolone, metenolone, nandrolone, stanozolol, esters of testosterone, and trenbolone.   The substances taken orally included 4-dehydrochlormethyltestosterone, fluoxymesterone, mesterolone, metenolone, metandienone, oxandrolone, oxymetholone, and stanozolol.” | N/A | N/A | N/A | N/A | N/A |
| [**Angell, 2012**](https://doi.org/10.1249/mss.0b013e3182358cb0) |  | Trenbolone (15)  Testosterone (13) Sustanon (9) Boldenone (7) Nandrolone (10) Methandrostenolone (8)  Drostanolone propionate (3) Oxandrolone (4) Stanozolol (3) | Trenbolone (IM)  Sustanon (IM)  Boldenone (IM)  Nandrolone (IM)  Methandrostenolone (IM)  Drostanolone propionate (IM)  Testosterone propionate (IM)  Testosterone cypionate (IM)  Nandrolone decanoate (IM)  Oxandrolone (Oral) Stanozolol (Oral)  (81,8% IM \| 18,2% Oral) | Testosterone propionate: 800 mg Testosterone cypionate: 500 mg Nandrolone decanoate: 600 mg Stanozolol: 350 mg | N/A | N/A |
