# Supplementary material for: Duration‐Dependent Cardiac Effects of Anabolic‐Androgenic Steroid Use in Strength‐Trained Athletes: A Systematic Review and Meta‐Analysis
Source: Echocardiography. 2026 Aug 31;43(9):e70611. doi: 10.1111/echo.70611 (PMC13528975; doi:10.1111/echo.70611)
Supplement: Supplementary file 4 [file ECHO-43-e70611-s004.docx]

**Supplementary Material 5**

**Risk of bias summary for non-randomized studies (ROBINS-I).**

| **Study** | **Bias due to confounding** | **Bias in selection of participants** | **Bias in classification of interventions** | **Bias due to deviations from intended interventions** | **Bias due to missing data** | **Bias in measurement of outcomes** | **Bias in selection of the reported result** | **Overall risk of bias judgement** |
| --- | --- | --- | --- | --- | --- | --- | --- | --- |
| Abdullah, 2023 | Moderate | Moderate | Moderate | Low | Low | Low | Low | Moderate |
| Akçakoyun, 2014 | Moderate | Moderate | Moderate | Low | Low | Low | Low | Moderate |
| Alizade, 2016 | Moderate | Moderate | Moderate | Low | Low | Low | Moderate | Moderate |
| Angell, 2012 | Moderate | Moderate | Moderate | Low | Low | Low | Moderate | Moderate |
| Baggish, 2010 | Moderate | Moderate | Moderate | Low | Low | Low | Moderate | Moderate |
| Baggish, 2017 | Moderate | Moderate | Moderate | Moderate | Low | Low | Moderate | Moderate |
| Bigi, 2021 | Moderate | Moderate | Moderate | Low | Low | Low | Moderate | Moderate |
| Buhl, 2025 | Moderate | Moderate | Low | Low | Low | Low | Low | Moderate |
| Castro, 2025 | Moderate | Moderate | Moderate | Low | Low | Low | Moderate | Moderate |
| D’Andrea, 2021 | Moderate | Moderate | Moderate | Low | Low | Low | Low | Moderate |
| De Piccoli, 1991 | Moderate | Moderate | Moderate | Low | Low | Low | Low | Moderate |
| Deligiannis, 1992 | Moderate | Moderate | Moderate | Low | Low | Moderate | Moderate | Moderate |
| Di Bello, 1999 | Moderate | Moderate | Moderate | Low | Low | Low | Moderate | Moderate |
| Dickerman, 1997 | Moderate | Moderate | Moderate | Low | Low | Low | Moderate | Moderate |
| Francis Ribeiro de Souza, 2021 | Moderate | Moderate | Moderate | Low | Low | Low | Low | Moderate |
| Fyksen, 2025 | Moderate | Moderate | Low | Low | Low | Low | Low | Moderate |
| Grandperrin, 2022 | Moderate | Moderate | Moderate | Low | Low | Low | Low | Moderate |
| Grandperrin, 2023 | Moderate | Moderate | Moderate | Low | Low | Low | Low | Moderate |
| Haji, 2022 | Moderate | Moderate | Moderate | Low | Low | Low | Low | Moderate |
| Hajimoradi, 2012 | Moderate | Moderate | Moderate | Low | Low | Low | Low | Moderate |
| Hammoud, 2023 | Moderate | Moderate | Moderate | Low | Low | Low | Low | Moderate |
| Hartgens, 2003 - 1 | Moderate | Moderate | Moderate | Low | Low | Moderate | Moderate | Moderate |
| Hartgens, 2003 - 2 | Moderate | Moderate | Moderate | Low | Low | Moderate | Moderate | Moderate |
| Ilié, 2014 | Moderate | Moderate | Moderate | Low | Low | Low | Low | Moderate |
| Kahnouji, 2022 | Moderate | Moderate | Moderate | Low | Low | Low | Low | Moderate |
| Kasikcioglu, 2009 | Moderate | Moderate | Moderate | Low | Low | Low | Moderate | Moderate |
| Kaya, 2019 | Moderate | Moderate | Moderate | Low | Low | Low | Low | Moderate |
| Kouidi, 2021 | Moderate | Moderate | Moderate | Low | Low | Low | Low | Moderate |
| Krieg, 2007 | Moderate | Moderate | Moderate | Low | Low | Low | Moderate | Moderate |
| Montisci, 2010 | Moderate | Moderate | Moderate | Low | Low | Low | Low | Moderate |
| Neto, 2016 | Moderate | Moderate | Moderate | Low | Low | Low | Moderate | Moderate |
| Nottin, 2006 | Moderate | Moderate | Moderate | Low | Low | Low | Low | Moderate |
| Palatini, 1996 | Moderate | Moderate | Moderate | Low | Low | Low | Low | Moderate |
| Place, 2023 | Moderate | Moderate | Moderate | Low | Low | Low | Moderate | Moderate |
| Sachtleben, 1993 | Moderate | Moderate | Moderate | Low | Low | Moderate | Moderate | Moderate |
| Sader, 2001 | Moderate | Moderate | Low | Low | Low | Low | Low | Moderate |
| Salke, 1985 | Moderate | Moderate | Moderate | Low | Low | Moderate | Moderate | Moderate |
| Thompson, 1992 | Moderate | Moderate | Moderate | Low | Low | Low | Low | Moderate |
| Urhausen, 2004 | Moderate | Moderate | Moderate | Low | Low | Low | Low | Moderate |
| Yeater, 1996 | Moderate | Moderate | Moderate | Low | Low | Moderate | Low | Moderate |

ROBINS-I tool: Sterne et al. BMJ 2016;355:i4919.

1. LVEF Funnel Plot and Egger’s Test.

2. GLS Funnel Plot and Egger’s Test.

3. E/A Funnel Plot and Egger’s Test.

4. E/e’ Funnel Plot and Egger’s Test.
