# Supplementary material for: Duration‐Dependent Cardiac Effects of Anabolic‐Androgenic Steroid Use in Strength‐Trained Athletes: A Systematic Review and Meta‐Analysis
Source: Echocardiography. 2026 Aug 31;43(9):e70611. doi: 10.1111/echo.70611 (PMC13528975; doi:10.1111/echo.70611)
Supplement: Supplementary file 5 [file ECHO-43-e70611-s005.docx]

**Supplementary Material 1**

**Search Strategy:** ("Anabolic Androgenic Steroid" OR "anabolic steroids" OR "androgenic steroids") AND (cardiovascular OR Echocardiography OR echocardiogram OR "Cardiac magnetic resonance" OR "cardiac MRI" OR "left ventricle" OR "ejection fraction" OR "wall thickness" OR "acute myocardial infarction" OR "percutaneous coronary intervention" OR "coronary artery bypass" OR "thromboembolism" OR "stroke" OR "ischemic stroke" OR "arrhythmia" OR "cardiomyopathy" OR "heart failure" OR HF OR "cardiac arrest").
